# Supplementary material for: Mutation Rate Variation is a Primary Determinant of the Distribution of Allele Frequencies in Humans
Source: PLoS Genet. 2016 Dec 15;12(12):e1006489. doi: 10.1371/journal.pgen.1006489 (PMC5157949; doi:10.1371/journal.pgen.1006489)
Supplement: S1 text — (PDF) [file pgen.1006489.s001.pdf]

Supplementary materials for: Mutation rate variation is a primary determinant of the distribution of allele frequencies in humans

Arbel Harpak[1,*,+], Anand Bhaskar[2,*] and Jonathan K. Pritchard[1,2,3]

[1] Department of Biology, Stanford University, Stanford, California, United States of America

[2] Department of Genetics, Stanford University, Stanford, California, United States of America

[3] Howard Hughes Medical Institute

[*] These authors contributed equally to this work

[+] Email: arbelh@stanford.edu

Table of Contents

Supplementary Notes

1. **Correlation of mutation rate and fraction of rare variants.** **3**
2. **Subsampling the SFS of mutation types.** **3**
3. **Treatment of reference allele in primate as fixed.** **4**
4. **The rate of ancestral polymorphisms under complex demographic histories.** **5**
5. **Testing for mutation-type-specific effects of chromatin state on the SFS.** **7**
6. **Unaccounted mutation rate variation.** **8**
7. **Mutation rate distribution at substituted-species sites.** **8**
8. **Effects of sequence context on the cSFS.** **14**

Supplementary Figures

**Figure S1:**	**Alternative conditioning of the human SFS on primate divergence patterns.**	**18**
**Figure S2:**	**Mutational composition of cSFS.**	**19**
**Figure S3:**	**The probability of an ancestral polymorphism as a function of the effective population size prior to the out-of-Africa event.**	**20**
**Figure S4:**	**SFS subsampling and the effect of mutation rate.**	**21**
**Figure S5:**	**Recombination rate and the skewness of the SFS.**	**22**
**Figure S6:**	**Distributions of mutation rates at substituted-species sites, in a constant molecular-clock model.**	**23**
**Figure S7:**	**cSFS species trend stratified by mutation type.**	**24**
**Figure S8:**	**Effects of sequence context at nonsynonymous sites.**	**25**
**Figure S9:**	**Epistatic effects of sequence context – amino acid context divergence.**	**26**
**Figure S10:**	**Epistatic effects of sequence context – distance from nearest substitution.**	**27**
**Figure S11:**	**Branch length parameters for the model of mutation rate distributions at substituted-species sites.**	**28**
**Figure S12:**	**Depletion of rare variants is correlated with relatedness to substituted species including CpG transitions.**	**29**

Correlation of mutation rate and fraction of rare variants

For the analysis on the effect of mutation rates on the SFS (Figure 3 and Figure 4) we wished to polarize sites into ancestral and derived states. We used agreement with both the chimpanzee and the gorilla reference to call an allele ancestral. Sites without an agreement between the chimpanzee and gorilla references were excluded from any further analysis involving derived allele frequencies. This left us with 1,859,979 intronic SNPs. Additionally, we downloaded the list of de-novo germline mutations found in the supplementary material of Kong et al [1]. For each C>T or G>A mutation, the upstream and downstream basepair from the hg18 build of the human reference genome were downloaded from the UCSC genome browser and used to ascertain whether the mutation is a CpG>TpG mutation (on either of the strands). We then used the estimate of mean genome-wide point mutation rate ($1.2 \cdot 10^{-8}$ mutations per bp per generation) to translate mutation-type counts into rates. Finally, for the estimate of the SFS in Europeans for each mutation type, we used the derived allele frequencies in the non-Finnish-European subsample of ExAC, as the Finnish population is presumed to be heavily bottlenecked and is overrepresented in the ExAC dataset [2].

Subsampling the SFS of mutation types

We sought to examine how sample size affects the sample SFS and the trend in SFS across mutation types. To do this, we computed the expected SFS over random subsamples of a given size from the SFS observed in the ExAC sample. We categorized intronic variants in non-Finnish Europeans into transversions, non-CpG transitions and CpG transitions. For each biallelic variant with minor allele count K and sample size M, the probability that a random subsample of size n contains j minor allele copies is given by

$$\frac{\binom{K}{j}\binom{M-K}{n-j}}{\binom{M}{n}} + (1-\delta_{j,n-j})\frac{\binom{K}{n-j}\binom{M-K}{j}}{\binom{M}{n}},$$

where $\delta_{a,b} = 1$ if $a = b$, and $\delta_{a,b} = 0$ otherwise. The probability that this site would constitute a rare variant in the subsample of size $n > 4$ is the probability of having 1 or 2 minor allele copies,

$$\frac{\binom{K}{1}\binom{M-K}{n-1} + \binom{K}{n-1}\binom{M-K}{1} + \binom{K}{2}\binom{M-K}{n-2} + \binom{K}{n-2}\binom{M-K}{2}}{\binom{M}{n}}. \qquad (1)$$

However, the site would only be polymorphic in the subsample of size n with probability

$$1 - \frac{\binom{K}{0}\binom{M-K}{n}}{\binom{M}{n}} - \frac{\binom{K}{n}\binom{M-K}{0}}{\binom{M}{n}}. \qquad (2)$$

To get the expected fraction of segregating sites in the original sample that are rare for a subsample of size n, we sum over the probability terms (1) of a site being rare in the subsample, and divide by the sum over the probability terms (2) of a site being polymorphic in the subsample.

Treatment of reference allele in primate as fixed

Throughout this work, we treated the observation of the human minor allele in the reference genome of a primate as evidence of an independent occurrence of the mutation and its fixation in that primate. However, several other scenarios are possible. For one, the human minor allele may not be fixed in the primate's lineage. We do not expect such cases to invalidate our results, since an allele that appears in the reference genome of a primate is likely to be of appreciable frequency in the primate population, (which also suggests that the allele is benign in the genomic context of the primate). Another option is that the human major allele is not fixed in the human-chimpanzee ancestral population (i.e., either the human minor allele is fixed or the polymorphism is ancient). In such cases the minor allele is more likely to be common in humans. This poses a concern for chimpanzee-substituted sites. Lastly, incomplete lineage sorting (ILS) can make the order of divergence times at a locus differ from that of the species [3,4]. ILS is expected to drive the chimpanzee-cSFS and gorilla-cSFS closer together. ILS is at least 1-2 orders of

magnitude less common for orangutan [5] and we therefore expect a small contribution of sites conferring to this evolutionary scenario for more distant species.

The rate of ancestral polymorphisms under complex demographic histories

In order to construct an upper bound on the probability of a human polymorphic site being ancestrally shared with another species, we consider the case of a neutral allele shared with chimpanzees. A polymorphism observed in the human sample at the current time is an ancestral polymorphism at the time of the human-chimpanzee split only if there are at least two lineages ancestral to the human sample that have not yet coalesced at the human-chimpanzee split time. We will assume that the effective population size prior to the out-of-Africa (OOA) event is constant and denoted by $\mathcal{N}$ haploids. This population size $\mathcal{N}$ will also be used for rescaling time into coalescent units. In what follows, the time parameters will be measured in coalescent units (i.e. in units of $\mathcal{N}$ generations). If we let $N(t)$ denote the time-varying effective population size function, then the cumulative coalescence rate up to time τ, $\Omega(\tau)$ is given by,

$$\Omega(\tau) = \int_0^\tau \frac{\mathcal{N}}{N(t)} dt \, .$$

If we let $A_n(t)$ denote the number of genealogical ancestors at time t of a sample of size n randomly drawn at time 0, we can in theory calculate the exact probability $\mathbb{P}(A_n(t) \geq 2)$ of having at least two lineages ancestral to the sample of size n at some time t in the past. However, the known closed-form formulae for computing the probabilities $\mathbb{P}(A_n(t) = m)$ in the ancestral process can exhibit numerical stability issues due to summation of terms of alternating signs, and we will instead use the first two moments of the ancestral process to give an upper bound on $\mathbb{P}(A_n(t) \geq 2)$. In particular, the expectation and variance of $A_n(t)$ can be computed numerically stably ([6], see Supplementary Information, equations 10 and 11) by

$$E[A_n(t)] = \sum_{i=1}^{n} e^{-\binom{i}{2}\Omega(t)}(2i-1)\frac{(n)_{i\downarrow}}{(n)_{i\uparrow}} ,$$

$$Var[A_n(t)] = \sum_{i=1}^{n} e^{-\binom{i}{2}\Omega(t)}(2i-1)(i^2-i+1)\frac{(n)_{i\downarrow}}{(n)_{i\uparrow}} - E[A_n(t)]^2,$$

where for $n \in \mathbb{R}, i \in \mathbb{Z}_{\geq 0}$, the notation $(n)_{i\downarrow}$ and $(n)_{i\uparrow}$ represent the falling and rising factorials respectively, and are given by

$$(n)_{i\downarrow} = \begin{cases} n(n-1)\cdots\big(n-(i-1)\big) & \text{if } i \geq 1 \\ 1 & \text{if } i = 0 \end{cases},$$

$$(n)_{i\uparrow} = \begin{cases} n(n+1)\cdots\big(n+(i-1)\big) & \text{if } i \geq 1 \\ 1 & \text{if } i = 0 \end{cases}.$$

The probability of having two or more lineages surviving at time t that are ancestral to the sample of size n can then be bounded as follows,

$$\mathbb{P}(A_n(t) \geq 2) = \mathbb{P}(A_n(t) - \mathrm{E}[A_n(t)] \geq 2 - \mathrm{E}[A_n(t)]) \leq \mathbb{P}(|A_n(t) - \mathrm{E}[A_n(t)]| \geq 2 - \mathrm{E}[A_n(t)])$$

$$= \mathbb{P}\left(|A_n(t) - \mathrm{E}[A_n(t)]| \geq \frac{2 - \mathrm{E}[A_n(t)]}{\sqrt{Var[A_n(t)]}}\sqrt{Var[A_n(t)]}\right) \leq \frac{Var[A_n(t)]}{(2 - \mathrm{E}[A_n(t)])^2},$$

where the last inequality follows from Chebyshev's inequality assuming $\mathrm{E}[A_n(t)] < 2$. This assumption holds for the rest of our analysis. The sample size n is conservatively taken to be 125,000 haploids, slightly higher than the maximum sample size in ExAC. The human-chimpanzee split time T_s is roughly 250,000 generations or $t_s = T_s/\mathcal{N}$ coalescent units. Let T_o denote the time of the OOA event measured in generations and $t_o = T_o/\mathcal{N}$ be the corresponding time in coalescent units. We use $T_o = 4{,}000$ generations as a conservative estimate for the timing of the OOA event. We can split the integral for the cumulative coalesce rate $\Omega(t_s)$ up to the human-chimpanzee split time t_s into two terms, one accounting for the coalescence rate from the present until the OOA event, and another accounting for the more ancient coalescence rate as follows,

$$\Omega(t_s) = \int_0^{t_o} \frac{\mathcal{N}}{N(t)} dt + \int_{t_o}^{t_s} \frac{\mathcal{N}}{N(t)} dt = \int_0^{t_o} \frac{\mathcal{N}}{N(t)} dt + t_s - t_o \,.$$

For the first integral above, we can use any demographic model for the post-OOA demography. We computed the Chebyshev upper bound on the probability of having two or more surviving ancestral lineages at the human-chimpanzee split time t_s, as a function of $\mathcal{N}$. Figure S3 shows the upper bound for two cases — for the demographic model of Nelson et al. [7], and for a "worst-case" demography in which there are no coalescence events post-OOA, and thus the contribution of the first integral to $\Omega(t_s)$ is 0. The Nelson et al. model pertains to the European subpopulation alone, but we show it here just to illustrate that the bound for the "worst-case" demographic model is close to the bound attained under complex demographic models for recent, post-OOA human history.

Testing for mutation-type-specific effects of chromatin state on the SFS

To test whether chromatin states affect the fraction of rare variants in a mutation-type-specific manner, we used a logistic regression framework. We regressed the fraction of rare variants on the mutation types (6 non-CpG mononucleotide mutation types and CpG->TpG), chromatin states (13 chromHMM states) and interaction terms between mutation types and chromatin states. Let Y be a binary-valued random variable indicating whether a variant is rare, $\vec{\mu}$ be a vector of mutually exclusive indicator variables for each mutation type, and $\vec{c}$ be a vector of mutually exclusive indicator variables for the chromatin state of the site. We fitted the logistic regression model

$$logit\big(P(Y=1|\vec{\mu},\vec{c})\big) = \beta_0 + \vec{\beta}_\mu \cdot \vec{\mu} + \vec{\beta}_c \cdot \vec{c} + \overleftrightarrow{\beta}_{\mu,c} \cdot (\vec{\mu} \otimes \vec{c}),$$

where the parameter vectors $\beta_0, \vec{\beta}_\mu, \vec{\beta}_c$, and $\overleftrightarrow{\beta}_{\mu,c}$ were learned from the data. We tested whether any of the interaction terms $\overleftrightarrow{\beta}_{\mu,c}$ had significantly nonzero coefficients. All of the terms involving CpG->TpG

mutations had a significant effect ($p < 0.01$) whereas none of the interaction terms involving non-CpG mutation types had a significantly nonzero effect at the same significance level.

Unaccounted mutation rate variation

A potential explanation for the trend in Figure 6 could be additional variation in mutation rates that we have not accounted for. Much of this variation could potentially be explained away if we could get better estimates for the local mutation rate (for example, by considering further sequence context around the mutating nucleotide, epigenomic context, etc.). Sequence context has been demonstrated to explain fine-scale differences in mutation rates [8]. However, further partitioning of our data, even using 2 upstream and 2 downstream nucleotides of context, resulted in too few sites per mutation type to accurately estimate the respective SFS summaries. In addition, differences in trend between the mononucleotide correction (Figure 6B) and the 3-bp correction (Figure 6C) are small, suggesting that the majority of mutational effects have been controlled for. Nevertheless, the effects of sequence and genome context could be more far-reaching [9,10]; previous work has found substantial variation in mutational patterns among primates [11]. Hodgkinson and Eyre-Walker [12] found higher sharing of co-occurring polymorphisms between human and chimpanzee than between human and macaque. Relatedly, local mutation rates could also be more similar in more closely-related species: the more similar the genomic context of the focal site is, the more strongly mutation rates co-vary between the species, leading to more shared polymorphism patterns.

Mutation rate distribution at substituted-species sites

In this section, we derive a simple model of the distribution of mutation rates at substituted-species sites. We demonstrate that mutation rates at substituted-species sites are expected to be higher than those at

random sites. At the same time, this model predicts that the mutation rate distribution at substituted-species sites is insensitive to the identity of the substituted species. This latter prediction changes, however, when we augment the model with the notion of molecular clock rate variation in some substitution types.

We wish to find the distribution of mutation rates M at sites in the substituted species S category. Let $f(M)$ be the probability density function of the mutation rates at random sites, and $g(M|S)$ be the probability density function of the mutation rate at sites in the S category. By Bayes' rule we have,

$$g(M|S) = \frac{Pr(S|M)f(M)}{Pr(S)},$$

where $Pr(S)$ is the probability of a random site being a site in the S category, and $Pr(S|M)$ is the probability of a site with mutation rate M being a site in the S category.

Note that by the law of total probability,

$$g(M|S) = \frac{Pr(S|M)f(M)}{\int Pr(S|M')f(M')dM'}.$$

Let l_S denote the length in years of the external branch leading to species S in the primate phylogenetic tree (Figure S11), and let l denote the total branch length in years of the phylogenetic tree of the six primates under consideration,

$$l = \sum_S l_S + l_{baboon}.$$

Note that we define l_{baboon} differently from l_S for other species S: l_{baboon} includes both the external branch leading to baboon and the branch shared between baboon and macaque. This is done since a site is considered to be a substituted-macaque site if there is no substitution on the shared baboon-macaque lineage, whereas we do not consider substituted-baboon sites. Let t_{tot} denote the total branch length in

years of the genealogy of the human ExAC sample (we make the simplifying assumption that this branch length is the same at all sites).

The accumulation of substitutions along the primate phylogeny, and along the human sample genealogy, follows a Poisson process. Thus, a random site is an S substituted-species site if three independent events occur: (i) a substitution occurs on the external branch of length l_S leading to the non-human primate S, (ii) no substitution occurs elsewhere in the phylogeny, over a total length of $l - l_S$ years, and (iii) the site is polymorphic in the human sample due to a mutation occurring on any of the branches of the genealogy of the human sample, that are of total length t_{tot} years. Combining these three independent events, we get,

$$Pr(S|M) = (1 - \exp(-Ml_S)) \exp(-M(l - l_S)) (1 - \exp(-Mt_{tot})).$$

We now consider a low mutation rate regime, in which all mutation rates are small enough so that we can assume $Ml \ll 1$ throughout. In this case, we have that, up to the first order in Ml,

$$\Pr(S|M) \approx Ml_s(1 - \exp(-Mt_{tot})).$$

Plugging this result back to $g(M|S)$, it follows that,

$$g(M|S) = \frac{Pr(S|M)f(M)}{\int Pr(S|M')f(M')dM'} \approx \frac{M(1 - \exp(-Mt_{tot})f(M)}{E_{M'\sim f}[M'(1 - \exp(-M't_{tot}))]}.$$

Note that the above expression for $g(M|S)$ does not depend on the identity of S. Therefore, in this low mutation rate regime, we expect similar mutation rate distributions across the different substituted-species categories.

We now return to a more realistic setting in which we allow for hypermutability in some fraction of sites. We used a set of realistic parameters from the literature, and numerical integration of the functions derived above, to develop a theoretical expectation for $g(M|S)$. The external branch lengths, in years, of the primate phylogeny taken to be $l_{chimpanzee} = 3{,}000{,}000, l_{gorilla} = 5{,}500{,}000, l_{orangutan} = 11{,}000{,}000, l_{gibbon} = 16{,}800{,}000$, $l_{macaque} = 6{,}000{,}000$, and $l_{baboon} = 29{,}000{,}000$, based on

estimates by Carbone et al. [13] for gibbon and macaque, by Steiper et al. [14] for the baboon-macaque split, and by Prado-Martinez et al. [15] for great apes. Note that the macaque branch is short and the baboon branch is long. Again, this is because a site is considered to be a substituted-macaque site if there is no substitution on the shared baboon-macaque lineage, whereas we do not consider substituted-baboon sites. For the density $f(M)$ of mutation rates at random sites, we used a lognormal distribution similar to that estimated by Hodgkinson and Eyre-Walker [16], with a location parameter of $4.8 \cdot 10^{-10}$ mutations per year (corresponding to $1.2 \cdot 10^{-8}$ mutations per generation, assuming a constant generation time of 25 years across primates), and a scale parameter of 0.83. Namely,

$$M = 4.8 \cdot 10^{-10} \exp(0.83 \cdot Z),$$

where $Z \sim N(0,1)$. Finally, for the sum of branch lengths of the genealogy underlying the human sample, we set t_{tot} to the expected branch length under the Kingman coalescent for the human ExAC sample of 60,000 individuals under the European population demographic model inferred by Nelson et al. [7]. This resulted in $t_{tot} = 262{,}315{,}250$ years, which was computed using the analytic results implemented in the coalescent computation tools of Bhaskar et al. [6].

Figure S6 shows the density functions of four mutation rate distributions: the distribution at random sites $f(M)$, the densities $g(M|S = chimpanzee)$ and $g(M|S = gibbon)$ at substituted-chimpanzee and substituted-gibbon sites respectively, and the density $f_{poly}(M)$ at polymorphic sites, computed, following a similar derivation to that of $g(M|S)$, by,

$$f_{poly}(M) = \frac{(1 - \exp(-M t_{tot}))\, f(M)}{\int (1 - \exp(-M' t_{tot}))\, f(M') dM'}.$$

The results presented in Figure S6 ground the intuition that substituted-species sites are expected to have much higher mutation rates than random sites, or random polymorphic sites, because of the evidence for two independent occurrences of a mutation in these sites. However, the model predicts that the distributions of mutation rates across substituted-species categories of sites should be indistinguishable

from each other. As this prediction fails to explain the observed differences in cSFS and mutational composition, we sought to improve our model by considering various possible origins of mutations, and subsequent effects on the accumulation of these mutations across primates.

Whereas some mutation types, like CpG transitions, accumulate at a relatively constant yearly rate across different primate lineages, the rates of other mutation types depend on various life-history traits [17-19]. Notably, across mammals, the yearly rates of many mutation types are negatively correlated with generation times ("generation time effect", [19,20]).

At the same time, when we examined more closely the differences in mutational composition of substituted-species categories, we noticed a possibly-related trend: while the relatedness of the substituted-species correlates well with the fraction of CpG transitions, it appears to be uncorrelated with the non-CpG transition:transversion ratio. This observation led us to consider that the CpG enrichment trend is not due to their high mutation rate—because then one would expect a similar trend in non-CpG transition:transversion ratios, as transitions have a higher rate than transversions. Rather, we hypothesized the CpG enrichment is related to the different sensitivity of mutation types to the generation time effect.

Motivated by these considerations, we augmented our model by considering arising mutations to be a mixture of mutations whose yearly rate is either (i) sensitive ("*sen*"), or (ii) insensitive ("*ins*") to the generation time effect. Let a mutation at a random site be *sen* with probability π_{sen}, with a log-normally distributed rate with mean μ_{sen} per year and shape parameter 0.83, as before. Conversely, a mutation at a random site is *ins* with probability $1 - \pi_{sen}$ and its rate is log-normally distributed with a location parameter μ_{sen} per generation and shape parameter 0.83. *sen* mutations accumulate along branches with lengths $\{l_S\}_S$ as set in the previous section. For *ins* mutations, we will divide branch lengths by the respective generation times of the primate at the leaf of each external branch, $\{g_S\}_S$. In summary, we have that,

$$g(M|S) = \frac{\pi_{sen}\Pr(S|M, sen; \{\frac{l_S}{g_S}\})f(M; \mu_{sen}) + (1-\pi_{sen})Pr(S|M, ins; \{l_S\})f(M; \mu_{ins})}{Pr(S)},$$

and,

$$Pr(S|M, ins; \{l_S\}) = (1 - \exp(-Ml_S))\exp\big(-M(l - l_S)\big)(1 - \exp(-Mt_{tot})),$$

$$\Pr(S|M, sen; \{\frac{l_S}{g_S}\}) = \left(1 - \exp\left(-M\frac{l_S}{g_S}\right)\right)\exp\left(-M\left(l^{sen} - \frac{l_S}{g_S}\right)\right)\left(1 - \exp\left(-M\frac{t_{tot}}{g_{human}}\right)\right).$$

Finally, for $\mu_* \in \{\mu_{sen}, \mu_{ins}\}$ we have that,

$$M; \mu_* = \mu_* \exp(0.83 \cdot Z),$$

where $Z \sim N(0,1)$.

Based on estimates of primate generation times from the literature, we set the following generation times in years: $g_{human} = 29$ [21], $, g_{chimpanzee} = 25$ [22], $g_{gorilla} = 19$ [22], $g_{orangutan} = 27$ [23], $g_{gibbon} = 12$ [13], $g_{macaque} = 12$ [24], and $g_{baboon} = 11$ [25]. Generation times on internal branches were assumed to be 25 years, such that,

$$l^{sen} = \sum_S \frac{l_S}{g_S} + \frac{l_{baboon}}{25}.$$

Motivated by the example of CpG transitions (which have a much higher average rate and constitute approximately 20% of de-novo mutations [26]) as an *ins* mutation type, we set $\pi_{sen} = 0.8$, $\mu_{ins} = \frac{10^{-7}}{g_{human}}$ mutations per year and $\mu_{sen} = 10^{-8}$ mutations per generation.

Under this augmented model, lineages of primates with longer generation times have a higher fraction of *ins* substitutions among all substitutions, and will therefore tend to have higher mutation rates (Figure 5A). Using numerical integration, we computed the probability densities of the distribution of mutation rates at a random site, at polymorphic sites, at substituted-chimpanzee sites and at substituted-macaque sites (Figure 5B). The distribution of mutation rates at substituted-chimpanzee sites is skewed towards

higher mutation rates than at substituted-macaque sites, with corresponding means of $9.2 \cdot 10^{-8}$ mutations per human generation at substituted-chimpanzee sites, and $6.9 \cdot 10^{-8}$ mutations per human generation at substituted-macaque sites.

Effects of sequence context on the cSFS

One explanation for the residual trend observed in Figures 6B,C is that more-related species have, on average, more similar context on which the mutation occurs. We can interpret this residual trend as support for the following hypothesis: when the sequence context of the substituted species is similar to that of humans, the fixation of the human-minor allele in the substituted species suggests that the mutation is benign for humans. As sequence context diverges, epistatic effects may come into play and change the selective effect of the mutation [28,73,74].

We decided to investigate the effect of sequence context more directly, by examining a single substituted species at a time. In doing so, we removed the potential confounder of mutation rate differences across substituted-species categories. Within a single substituted species, we expect to observe a similar trend across sites with differing levels of context similarity. We hypothesized that the more diverged the sequence context in the substituted-species is from humans, the more deleterious the variant would tend to be in humans. The fraction of rare variants is therefore expected to increase with sequence context divergence. Importantly, the same trend is expected if wide sequence context is a key determinant of mutation rates [32,37]. A substituted-species site is expected to have a high mutation rate because it suggests two independent occurrences of the same mutation. If the mutations occurred, however, on diverged contexts, the evidence for mutability of the site in the human context is weaker. We would again expect a higher fraction of rare variants for diverged sequence contexts.

To test this, we computed the amino acid sequence-context divergence between human and gibbon at all substituted-gibbon nonsynonymous SNPs. As a measure of divergence, we computed the number of

mismatches between human and gibbon sequences in a window of 9 amino acids upstream and 9 downstream of the focal substituted-gibbon sites. We found that, as expected, the fraction of rare variants increased with sequence context divergence (univariate logistic regression $p = 3.3x10^{-13}$). As a control we examined the effect of context divergence in sites conserved across non-human primates. In these sites, the fraction of rare variants decreased with sequence context divergence from gibbon, consistent with higher regional mutation rates and lower constraint implied by higher sequence context divergence (Figure S8A). This trend reversal may suggest that sequence context has a comparable role to other regional determinants of the SFS—like regional mutation rates and regional selective constraint—in determining the trajectory of an allele. The same trends hold for all substituted-species categories (univariate logistic regression $p < 3.1x10^{-8}$ for each of the other four species, Figure S9). The trends also hold for a different measure of sequence context similarity, the distance to the nearest amino-acid substitution between humans and the substituted-species (t-test $p < 1.8x10^{-32}$ and see Figure S8B for gibbon, $p < 2.2x10^{-5}$ for Pearson correlation and see Figure S10 for each of the other species).

References

1. Kong A, Frigge ML, Masson G, Besenbacher S, Sulem P, et al. (2012) Rate of de novo mutations and the importance of father's age to disease risk. Nature 488: 471-475.
2. Lek M, Karczewski K, Minikel E, Samocha K, Banks E, et al. (2016) Analysis of protein-coding genetic variation in 60,706 humans. Nature: 030338.
3. Chen F-C, Li W-H (2001) Genomic divergences between humans and other hominoids and the effective population size of the common ancestor of humans and chimpanzees. The American Journal of Human Genetics 68: 444-456.
4. Patterson N, Richter DJ, Gnerre S, Lander ES, Reich D (2006) Genetic evidence for complex speciation of humans and chimpanzees. Nature 441: 1103-1108.
5. Hobolth A, Dutheil JY, Hawks J, Schierup MH, Mailund T (2011) Incomplete lineage sorting patterns among human, chimpanzee, and orangutan suggest recent orangutan speciation and widespread selection. Genome Research 21: 349-356.
6. Bhaskar A, Clark AG, Song YS (2014) Distortion of genealogical properties when the sample is very large. Proceedings of the National Academy of Sciences 111: 2385-2390.
7. Nelson MR, Wegmann D, Ehm MG, Kessner D, Jean PS, et al. (2012) An abundance of rare functional variants in 202 drug target genes sequenced in 14,002 people. Science 337: 100-104.
8. Aggarwala V, Voight BF (2016) An expanded sequence context model broadly explains variability in polymorphism levels across the human genome. Nature Genetics.
9. Siepel A, Haussler D (2004) Phylogenetic estimation of context-dependent substitution rates by maximum likelihood. Molecular Biology and Evolution 21: 468-488.
10. Michaelson JJ, Shi Y, Gujral M, Zheng H, Malhotra D, et al. (2012) Whole-genome sequencing in autism identifies hot spots for de novo germline mutation. Cell 151: 1431-1442.
11. Hwang DG, Green P (2004) Bayesian Markov chain Monte Carlo sequence analysis reveals varying neutral substitution patterns in mammalian evolution. Proceedings of the National Academy of Sciences of the United States of America 101: 13994-14001.
12. Hodgkinson A, Eyre-Walker A (2011) Variation in the mutation rate across mammalian genomes. Nature Reviews Genetics 12: 756-766.
13. Carbone L, Harris RA, Gnerre S, Veeramah KR, Lorente-Galdos B, et al. (2014) Gibbon genome and the fast karyotype evolution of small apes. Nature 513: 195-201.
14. Steiper ME, Young NM, Sukarna TY (2004) Genomic data support the hominoid slowdown and an Early Oligocene estimate for the hominoid–cercopithecoid divergence. Proceedings of the National Academy of Sciences of the United States of America 101: 17021-17026.
15. Prado-Martinez J, Sudmant PH, Kidd JM, Li H, Kelley JL, et al. (2013) Great ape genetic diversity and population history. Nature 499: 471-475.
16. Hodgkinson A, Ladoukakis E, Eyre-Walker A (2009) Cryptic variation in the human mutation rate. PLoS Biol 7: e1000027.
17. Moorjani P, Amorim CEG, Arndt PF, Przeworski M (2016) Variation in the molecular clock of primates. bioRxiv: 036434.
18. Kim S-H, Elango N, Warden C, Vigoda E, Soojin VY (2006) Heterogeneous genomic molecular clocks in primates. PLoS Genet 2: e163.
19. Gao Z, Wyman MJ, Sella G, Przeworski M (2016) Interpreting the dependence of mutation rates on age and time. PLoS Biol 14: e1002355.
20. Sayres MAW, Venditti C, Pagel M, Makova KD (2011) Do variations in substitution rates and male mutation bias correlate with life-history traits? A study of 32 mammalian genomes. Evolution 65: 2800-2815.

21. Fenner JN (2005) Cross-cultural estimation of the human generation interval for use in genetics-based population divergence studies. American Journal of Physical Anthropology 128: 415-423.
22. Langergraber KE, Prüfer K, Rowney C, Boesch C, Crockford C, et al. (2012) Generation times in wild chimpanzees and gorillas suggest earlier divergence times in great ape and human evolution. Proceedings of the National Academy of Sciences 109: 15716-15721.
23. Wich SA (2009) Orangutans: geographic variation in behavioral ecology and conservation: Oxford University Press.
24. De Rooij D, van Alphen M, van de Kant H (1986) Duration of the cycle of the seminiferous epithelium and its stages in the rhesus monkey (Macaca mulatta). Biology of Reproduction 35: 587-591.
25. Altmann J, Gesquiere L, Galbany J, Onyango PO, Alberts SC (2010) Life history context of reproductive aging in a wild primate model. Annals of the New York Academy of Sciences 1204: 127-138.
26. Kong A, Frigge ML, Masson G, Besenbacher S, Sulem P, et al. (2012) Rate of de novo mutations and the importance of father/'s age to disease risk. Nature 488: 471-475.

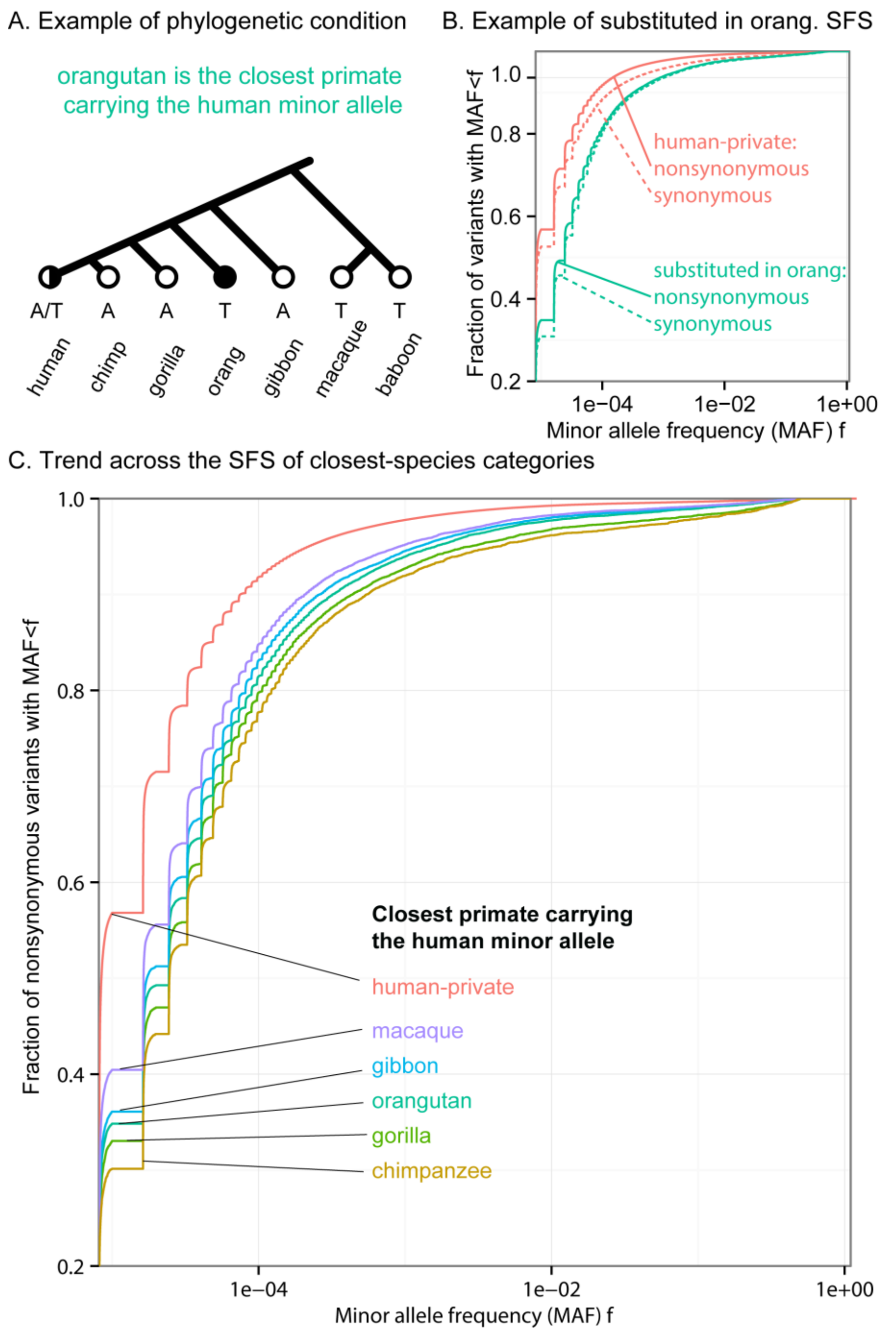

Fig S1. Alternative conditioning of the human SFS on primate divergence patterns. Changes in the human SFS as we condition on divergence patterns in primates. Here, we label the SFS by the primate most closely related to human carrying the human minor allele. **(A)** An example of the phylogenetic conditioning for sites in which orangutan is the closest primate which carries the human minor allele. **(B)** The cumulative distribution functions (CDF) of the SFS of sites substituted in orangutan, and the SFS of human-private mutations. The SFS of sites substituted in orangutan have a skew towards common alleles compared with human-private sites. **(C)** The more closely related the species with the substitution, the higher the skew of the SFS towards common variants (only nonsynonymous mutations shown).

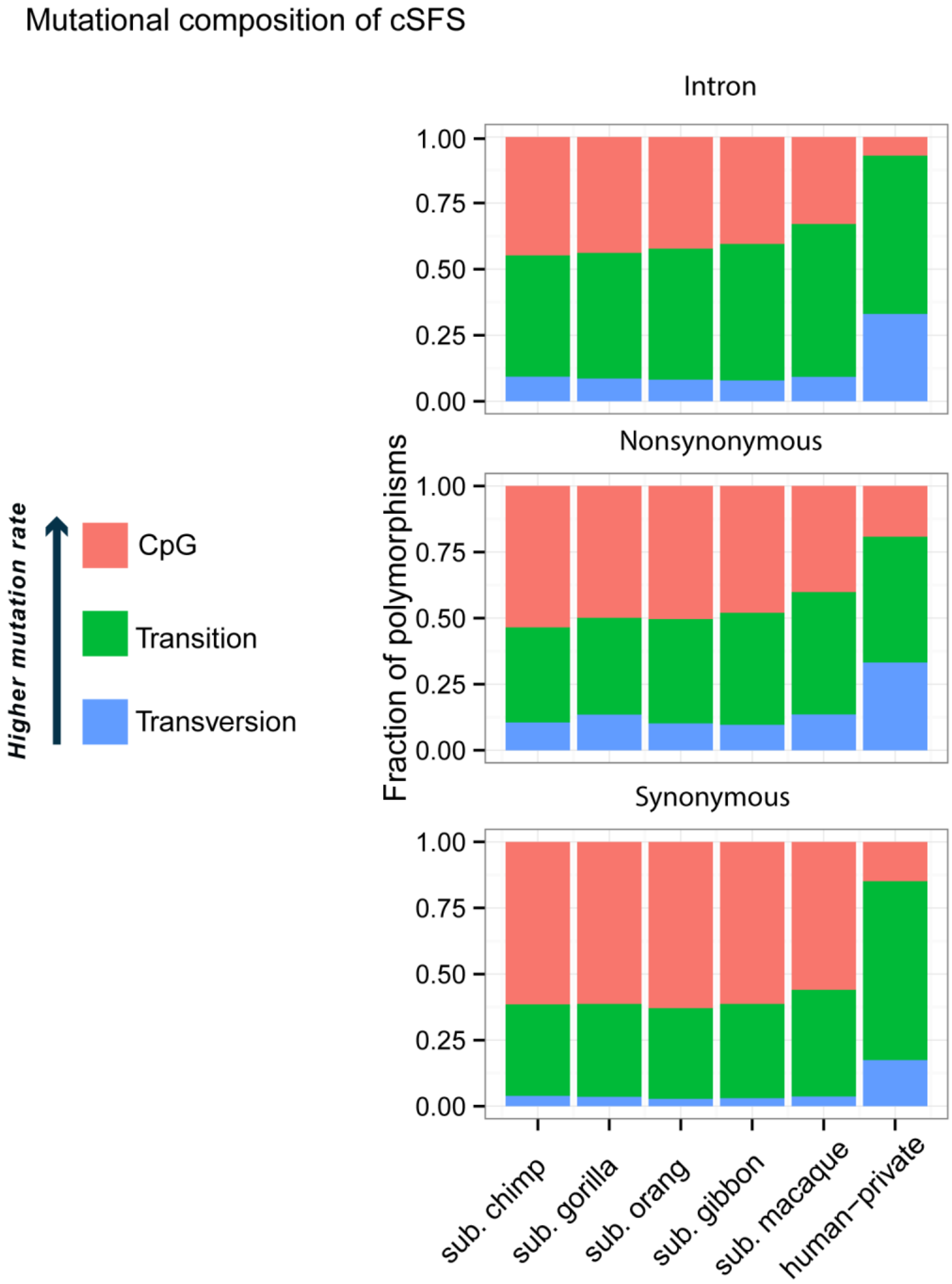

Fig S2. Mutational composition of cSFS. The fractions of three mutational categories associated with different mutability are shown for each substituted-species category. Substituted-species categories with lower fraction of rare variants are composed of more mutations conferring to high mutation rates.

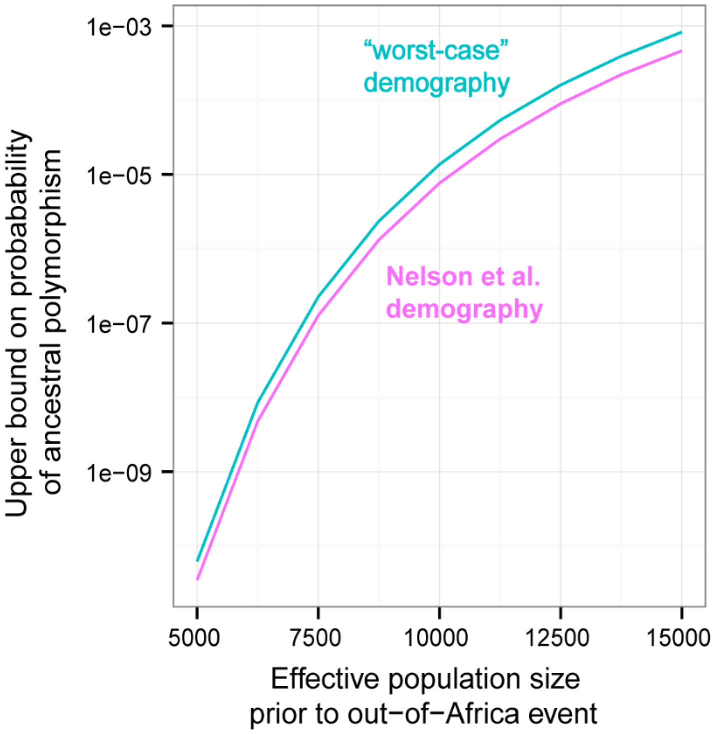

Fig S3. The probability of an ancestral polymorphism as a function of the effective population size prior to the out-of-Africa event. The y-axis shows an upper bound on the probability for a random polymorphism originating prior to the human-chimpanzee split time. Both models shown assume a constant effective population size prior to the out-of-Africa event (OOA); the pink line gives the upper bound for the Nelson et al. [7] demographic model after the OOA event, whereas the teal “worst-case” line gives the bound assuming no coalescence events occur between the OOA event and the present time.

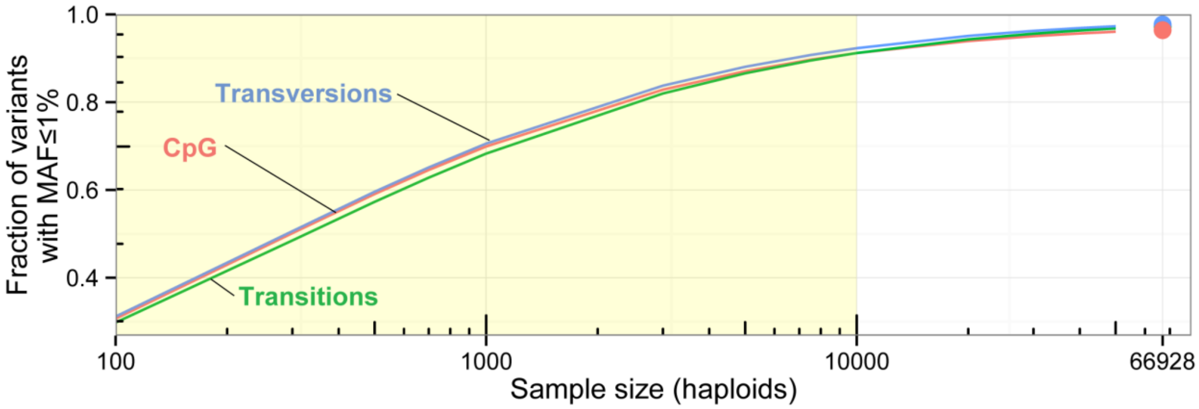

Fig S4. SFS subsampling and the effect of mutation rate. This figure summarizes the same analysis as in Figure 4B, with a summary of the fraction of variants with minor allele frequency (MAF) below or equal to 1%, instead of the fraction of rare variants. Dots show the fraction of variants with MAF ≤ 1% in the full sample SFS of the European population in ExAC. Lines show the expected fraction of variants with MAF ≤ 1% after subsampling to a smaller number of individuals. The trend between mutation types changes as the sample size varies (with an infliction point between CpG and non-CpG transitions marked by the border of the shaded region).

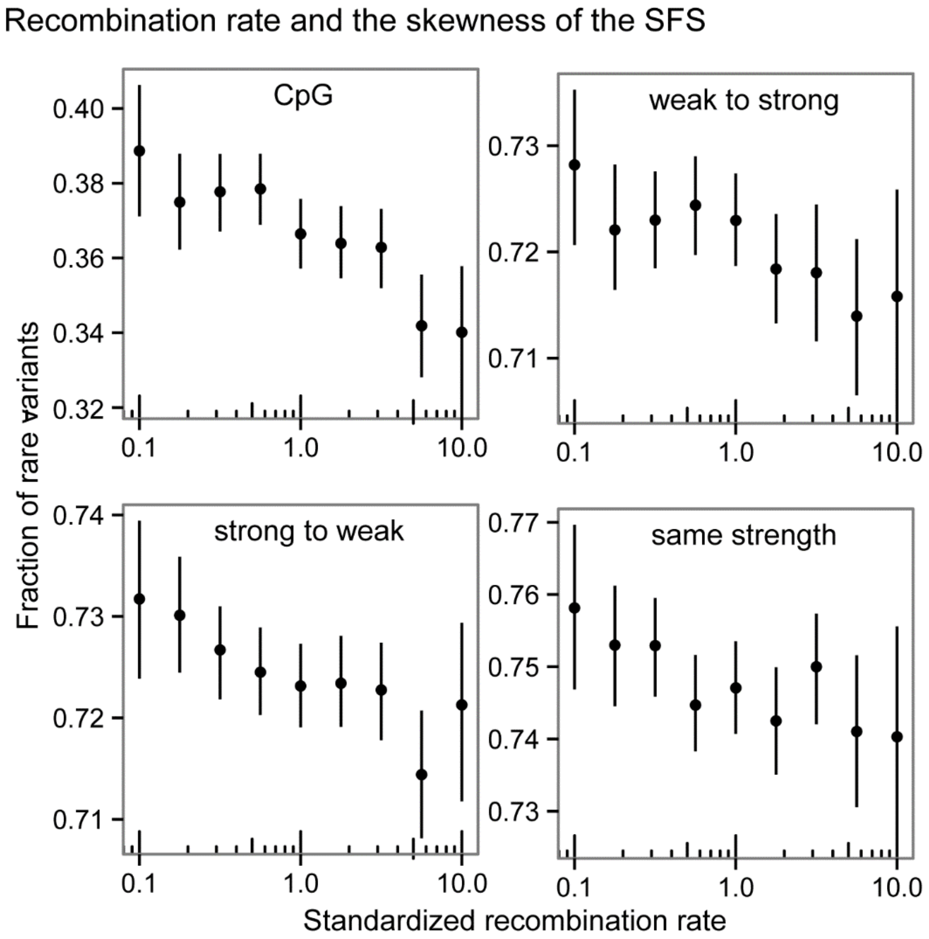

Fig S5. Recombination rate and the skewness of the SFS. Recombination rate is positively correlated with mutation rate, and is likely driving the negative correlation of recombination rate and the fraction of rare variants. The different panels demonstrate that mutations that are subject to biased gene conversion, as well as those that are not, exhibit a negative correlation between recombination rate and the fraction of rare variants. x-axis values are binned on a logarithmic scale, and are standardized to a genomewide mean.

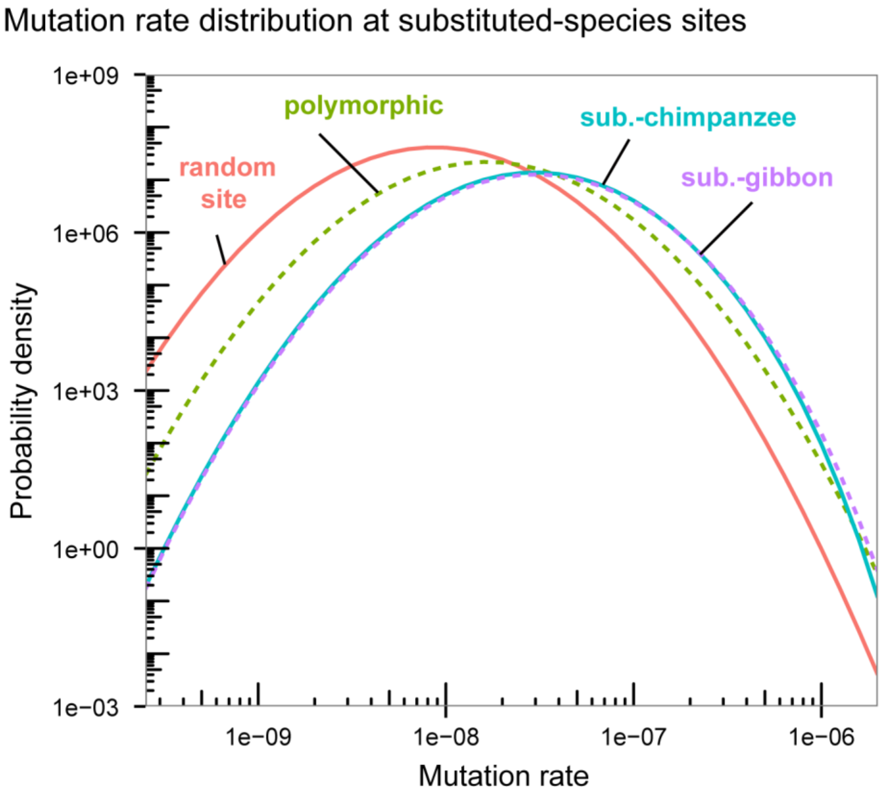

Fig S6. Distributions of mutation rates at substituted-species sites, in a constant molecular-clock model. These results were computed using a simple analytic model and a set of realistic parameters. At substituted-species sites, we expect a distribution skewed towards higher mutation rates compared to random sites, or to random polymorphic sites. However, the distribution of mutation rate changes only slightly across substituted species, as exemplified by the purple (substituted-gibbon) and teal (substituted-chimpanzee) lines.

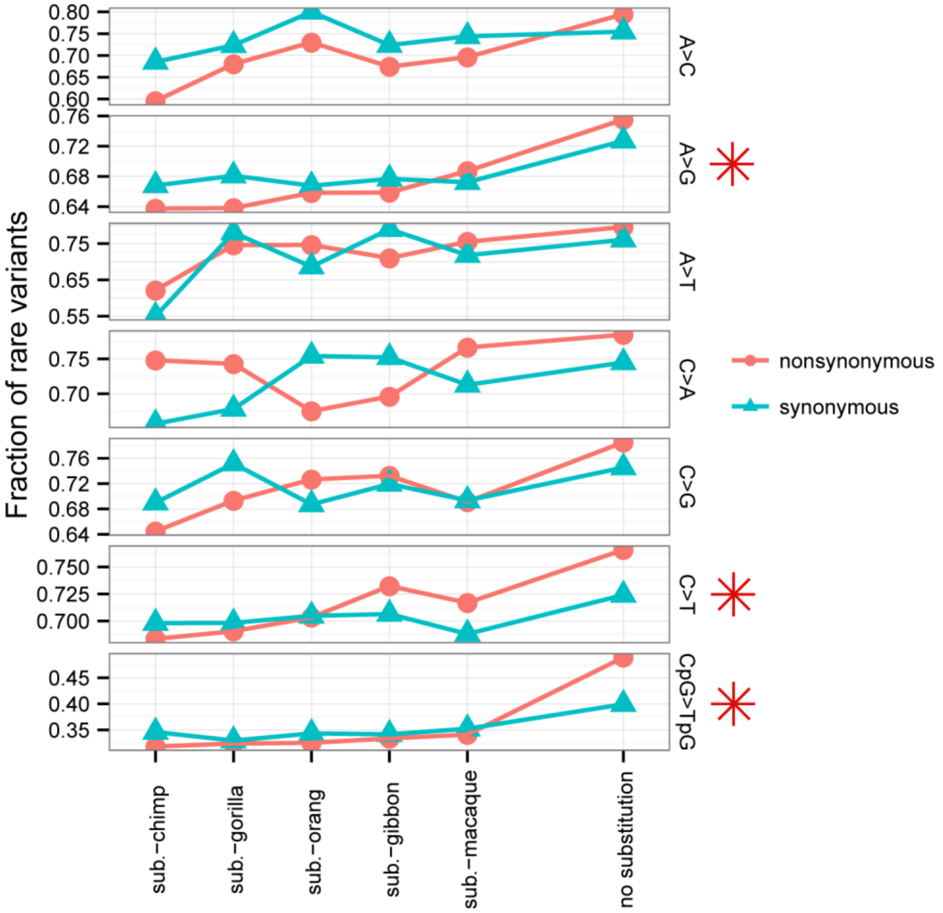

Fig S7. cSFS species trend stratified by mutation type. This figure summarizes the same analysis as in the inset of Figure 2C, stratified by non-CpG mononucleotide mutation types and CpG. Red stars denote nonsynonymous trends that exhibited significant spearman correlation at a significance level of 10%.

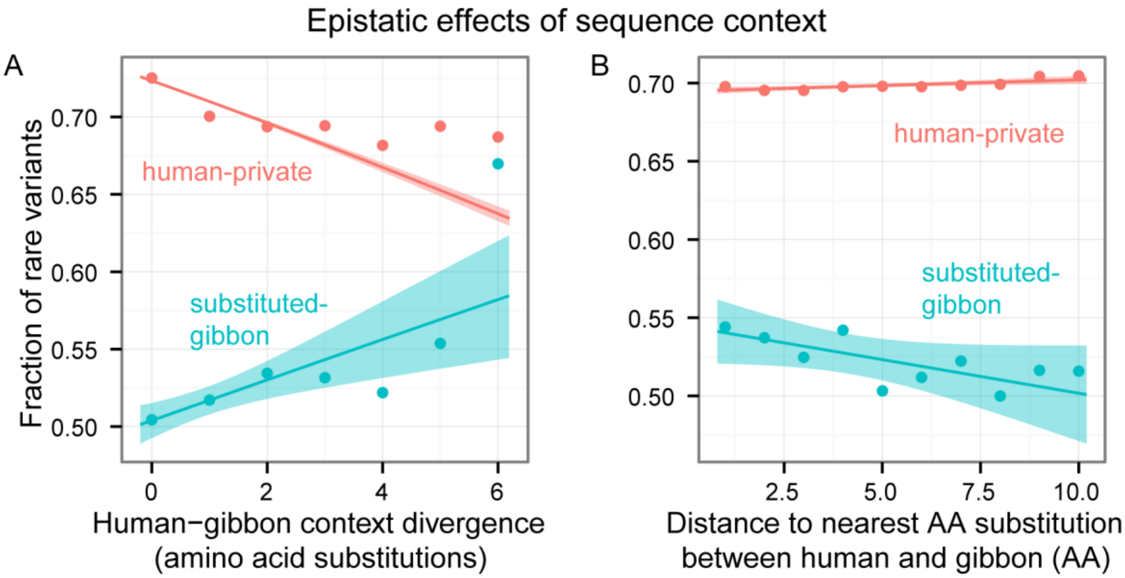

Figure S8. Effects of sequence context at nonsynonymous sites. Dots show means. Lines and shaded regions show simple logistic model fits to the data and associated confidence bands. The more diverged the sequence context in the substituted-species is from humans, the higher the fraction of rare variants. The two panels exhibit the same trend with different measures of sequence context divergence in a window of 9 residues upstream and 9 downstream of the SNP. In human-private sites, the trend is reversed: the fraction of rare variants decreased with sequence context divergence from gibbon, consistent with higher regional mutation rates and lower constraint implied by higher sequence context divergence.

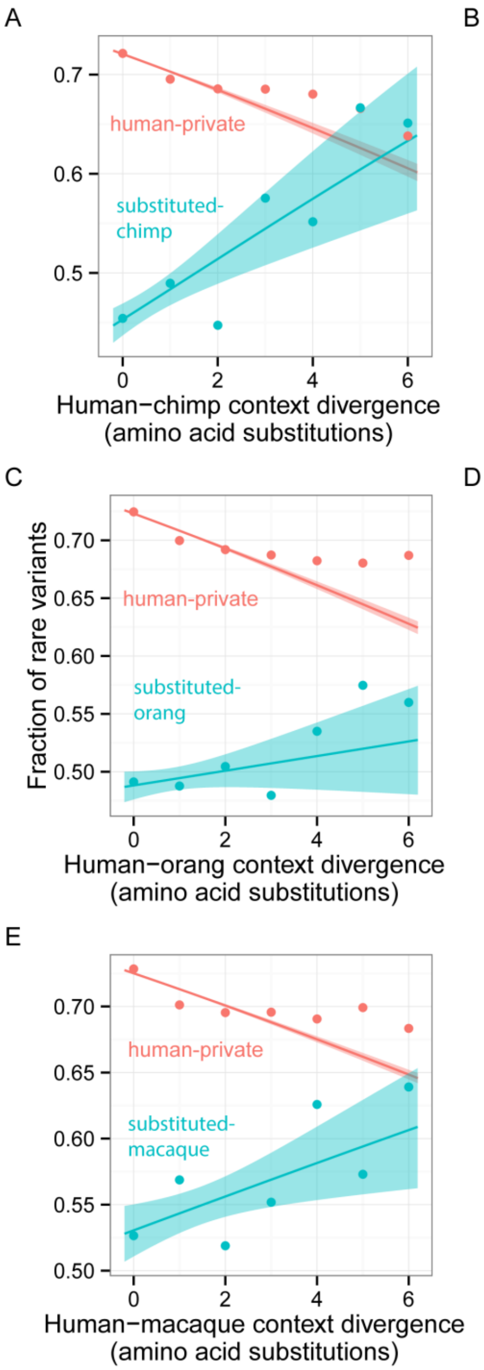

Fig S9. Epistatic effects of sequence context – amino acid context divergence. Dots show means. Lines and shaded regions show predictions of a simple logistic model fits to the data and their associated confidence bands. The more diverged the sequence context in the substituted-species is from humans, the higher the fraction of rare variants. Sequence context divergence is calculated as the number of amino acid substitutions in a window of 9 residues upstream and 9 downstream of the SNP. In human-private sites, the trend is reversed: the fraction of rare variants decreases with sequence context divergence from the substituted species, consistent with higher regional mutation rates and lower constraint implied by higher sequence context divergence.

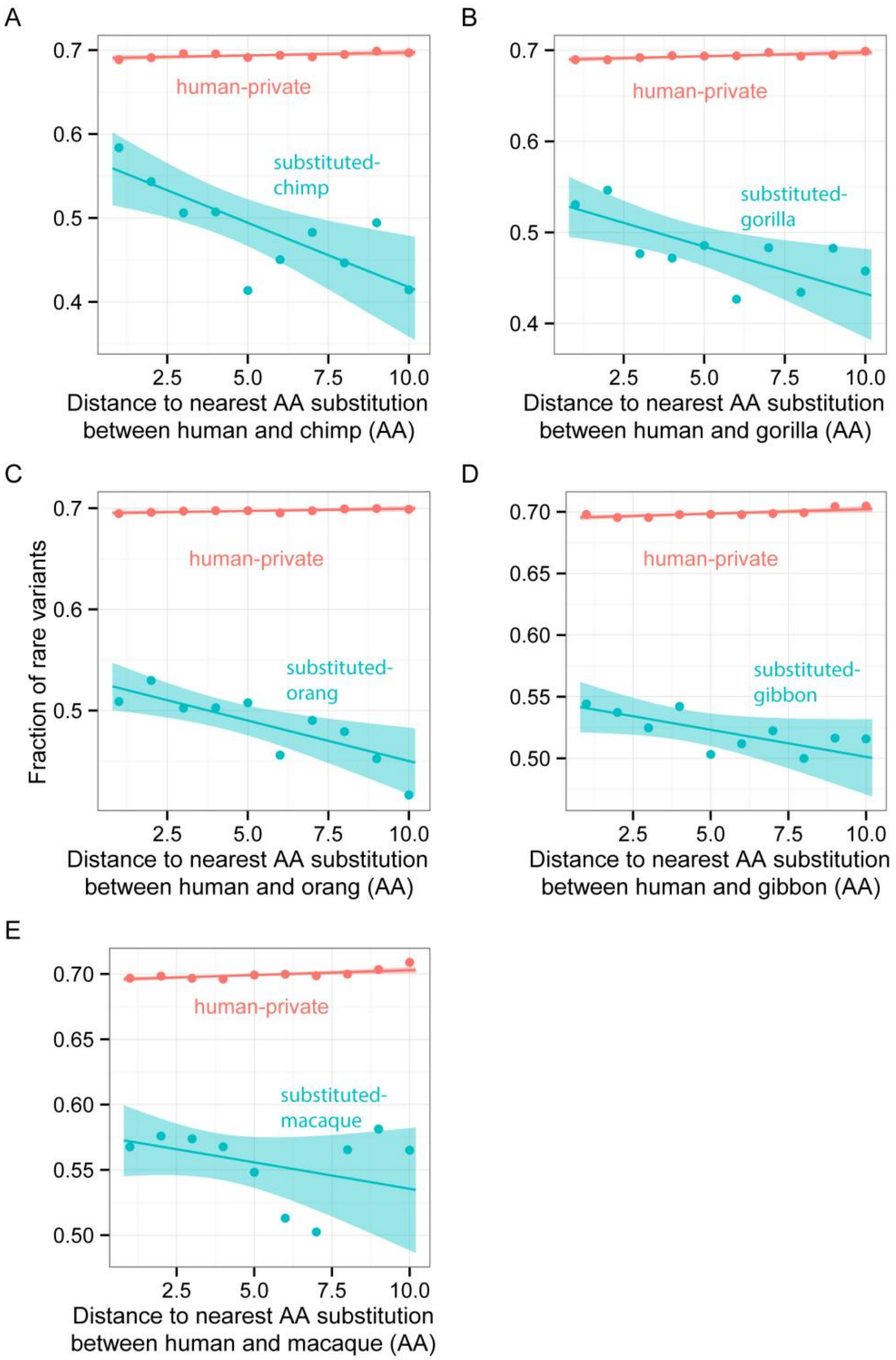

Fig S10. Epistatic effects of sequence context – distance from nearest substitution. Dots show means. Lines and shaded regions how predictions of a simple logistic model fits to the data and their associated confidence bands. The more diverged the sequence context in the substituted-species is from humans, the higher the fraction of rare variants. Sequence context divergence is calculated as the distance to the nearest amino acid substitutions in a window of 9 residues upstream and 9 downstream of the SNP.

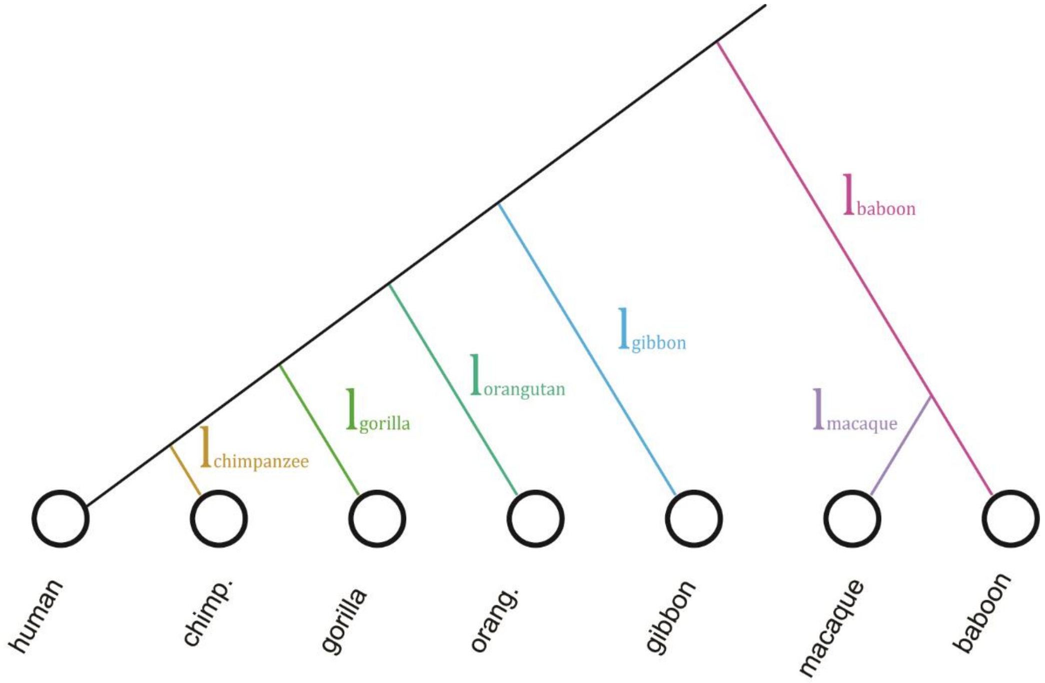

Fig S11. Branch length parameters for the model of mutation rate distributions at substituted-species sites.

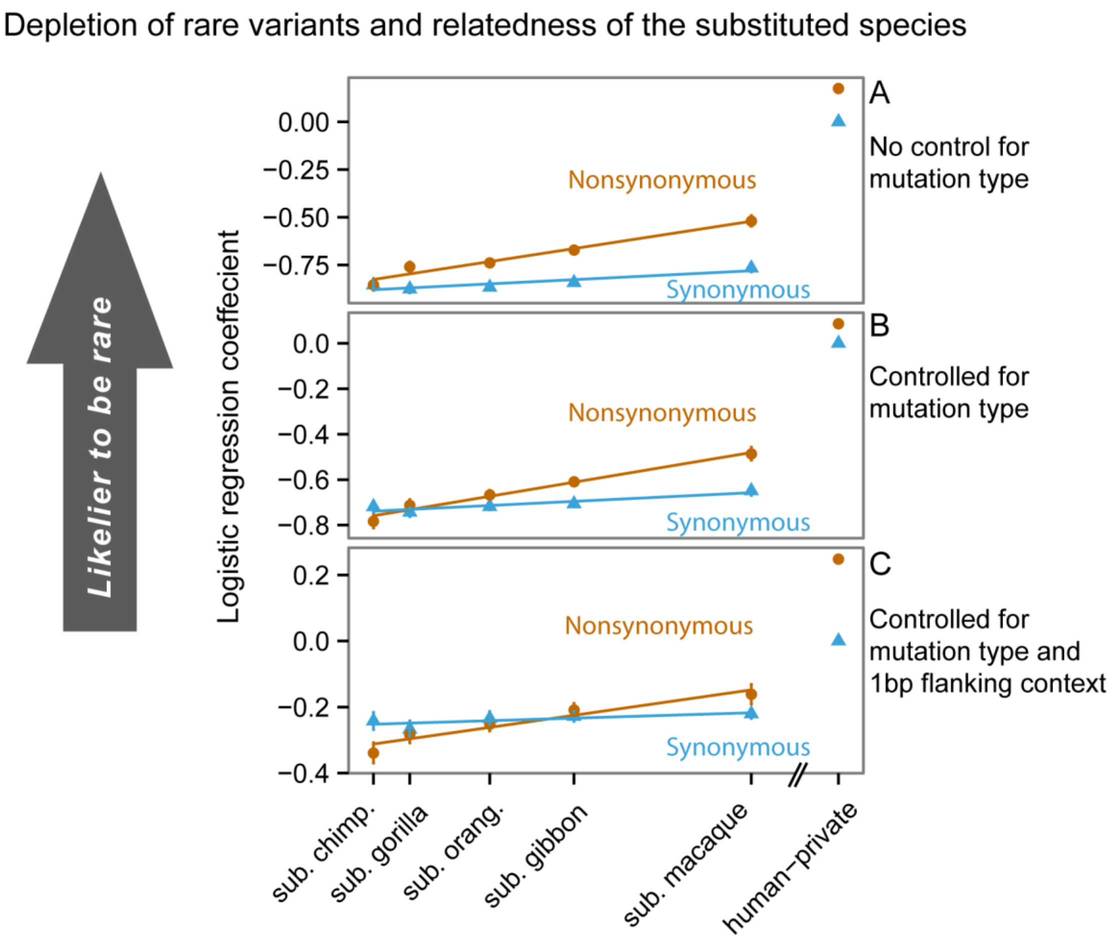

Figure S12. Depletion of rare variants is correlated with relatedness to substituted species including CpG transitions. Like Figure 6, this figure shows logistic regression coefficient estimates and their corresponding standard errors. Here, however, CpG transitions were included in the analysis. Substituted-species labels are spaced by their split times from humans. The lines are the least-squares line fitting the coefficients to the split times. **(A)** Estimates from a simple logistic regression to the substituted species. The trend is partly due to mutational composition differences between substituted-species categories. To test whether the trend is driven solely by mutational rate differences, we estimate coefficients in a model including the variation explained by **(B)** mononucleotide mutation type, and **(C)** combinations of focal mononucleotide mutations and upstream and downstream nucleotides. Even after controlling for mutational composition with these models, a significant trend persists for nonsynonymous variants.
