# Supplementary figures and images for: Mutation Rate Variation is a Primary Determinant of the Distribution of Allele Frequencies in Humans
Source: PLoS Genet. 2016 Dec 15;12(12):e1006489. doi: 10.1371/journal.pgen.1006489 (PMC5157949; doi:10.1371/journal.pgen.1006489)

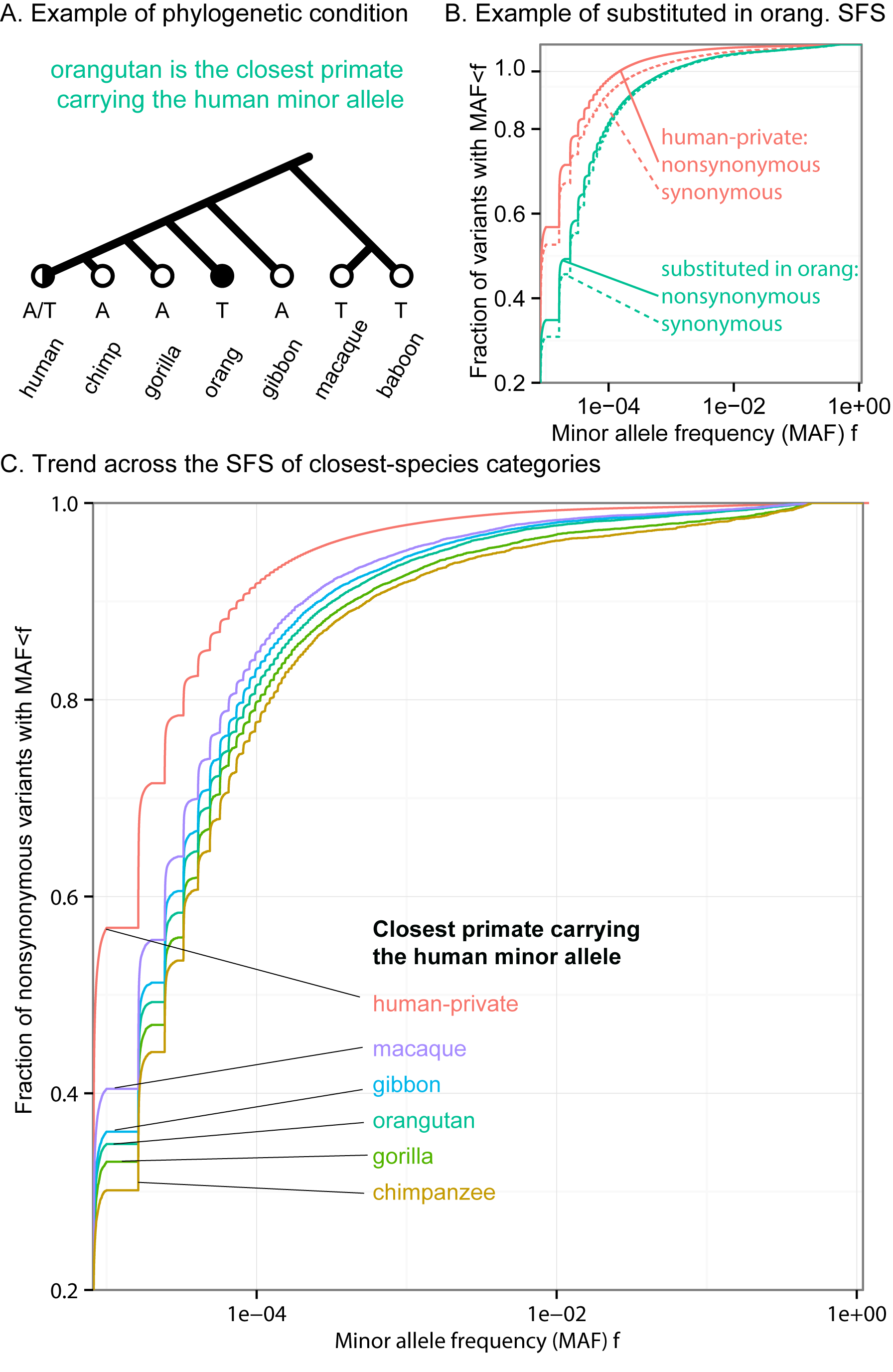

Supplement: S1 Fig — Changes in the human SFS as we condition on divergence patterns in primates. Here, we label the SFS by the primate most closely related to human carrying the human minor allele. (A) An example of the phylogenetic conditioning for sites in which orangutan is the closest primate which carries the human minor allele. (B) The cumulative distribution functions (CDF) of the SFS of sites substituted in orangutan, and the SFS of human-private mutations. The SFS of sites substituted in orangutan have a skew towards common alleles compared with human-private sites. (C) The more closely related the species with the substitution, the higher the skew of the SFS towards common variants (only nonsynonymous mutations shown). (TIF) [file pgen.1006489.s002.tif]

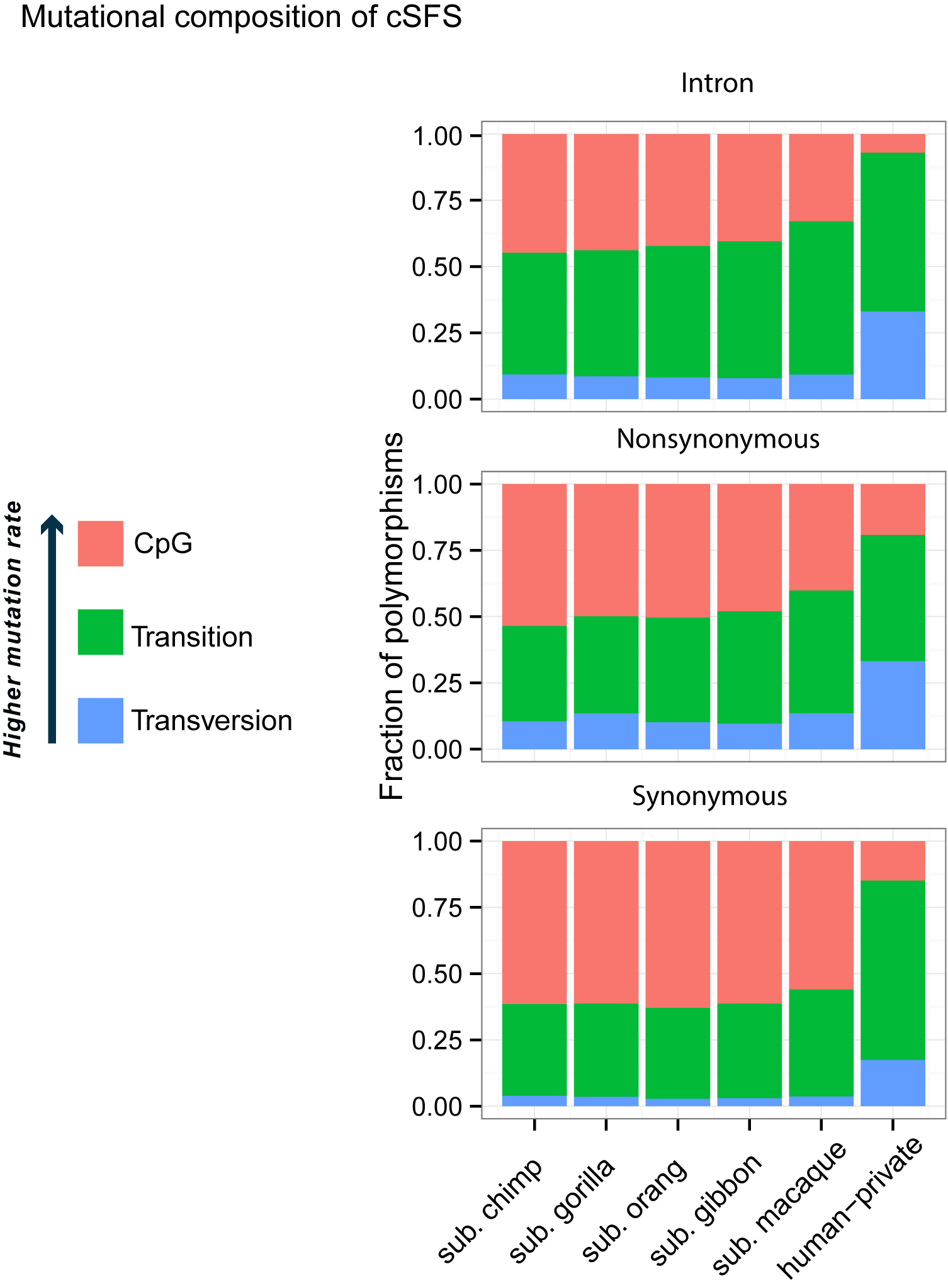

Supplement: S2 Fig — The fractions of three mutational categories associated with different mutability are shown for each substituted-species category. (TIF) [file pgen.1006489.s003.tif]

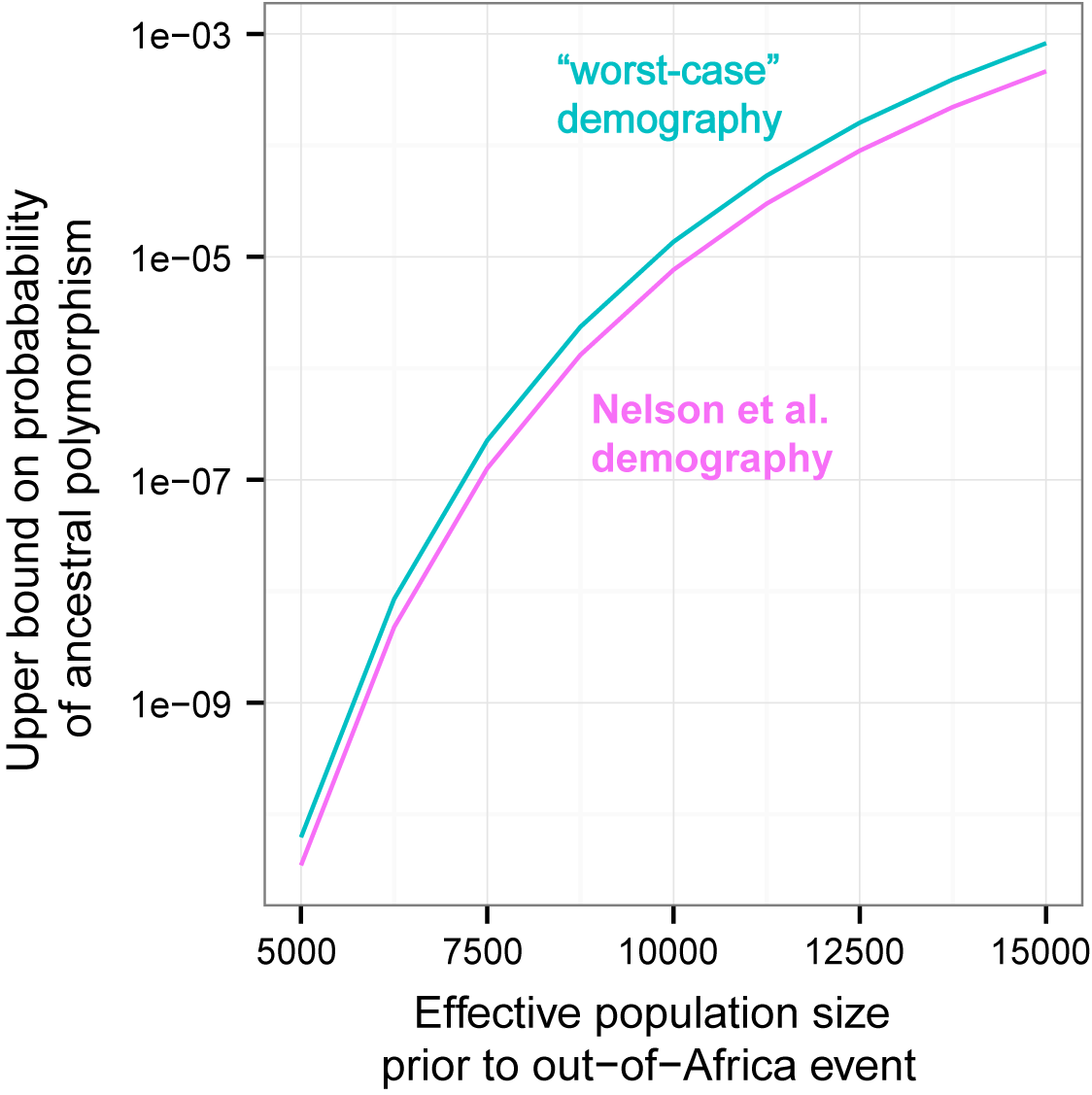

Supplement: S3 Fig — The y-axis shows an upper bound on the probability for a random polymorphism originating prior to the human-chimpanzee split time. Both models shown assume a constant effective population size prior to the out-of-Africa event (OOA); the pink line gives the upper bound for the Nelson et al. [7] demographic model after the OOA event, whereas the teal “worst-case” line gives the bound assuming no coalescence events occur between the OOA event and the present time. (TIF) [file pgen.1006489.s004.tif]

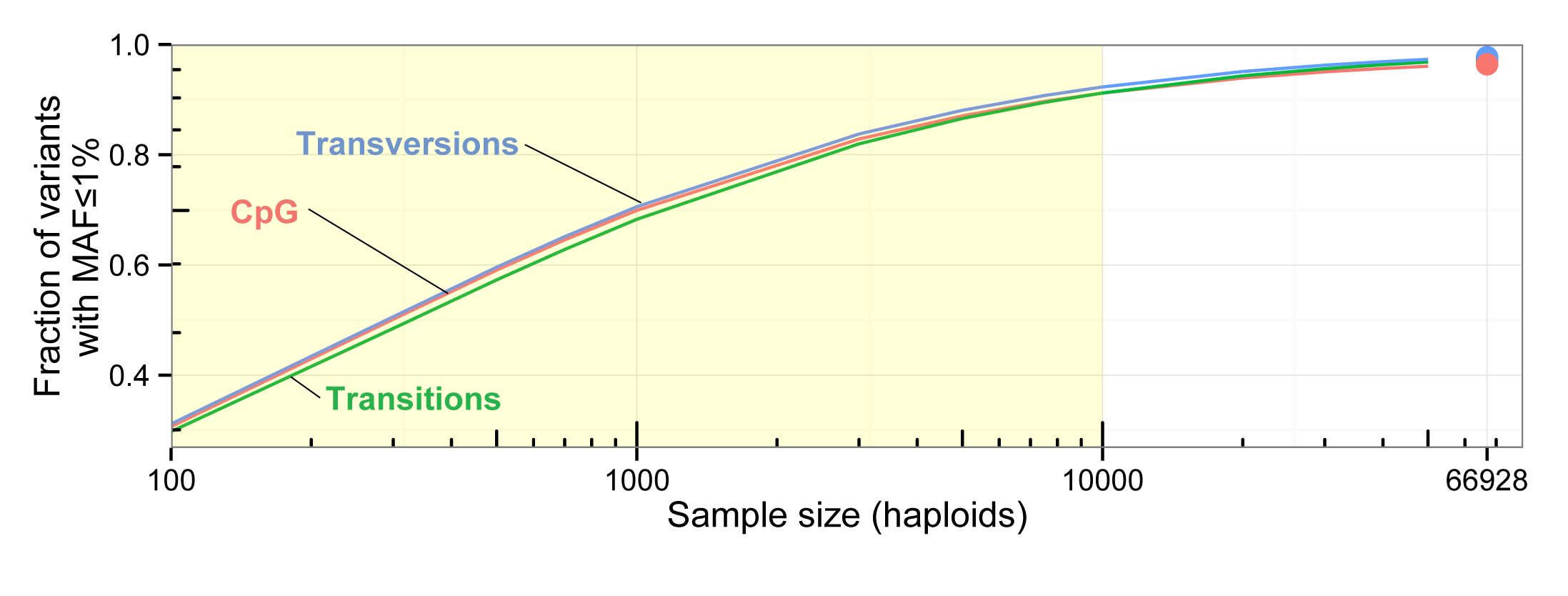

Supplement: S4 Fig — This figure summarizes the same analysis as in Fig 4B, with a summary of the fraction of variants with minor allele frequency (MAF) below or equal to 1%, instead of the fraction of rare variants. Dots show the fraction of variants with MAF ≤ 1% in the full sample SFS of the European population in ExAC. Lines show the expected fraction of variants with MAF ≤ 1% after subsampling to a smaller number of individuals. The trend between mutation types changes as the sample size varies (with an infliction point between CpG and non-CpG transitions marked by the border of the shaded region). (TIF) [file pgen.1006489.s005.tif]

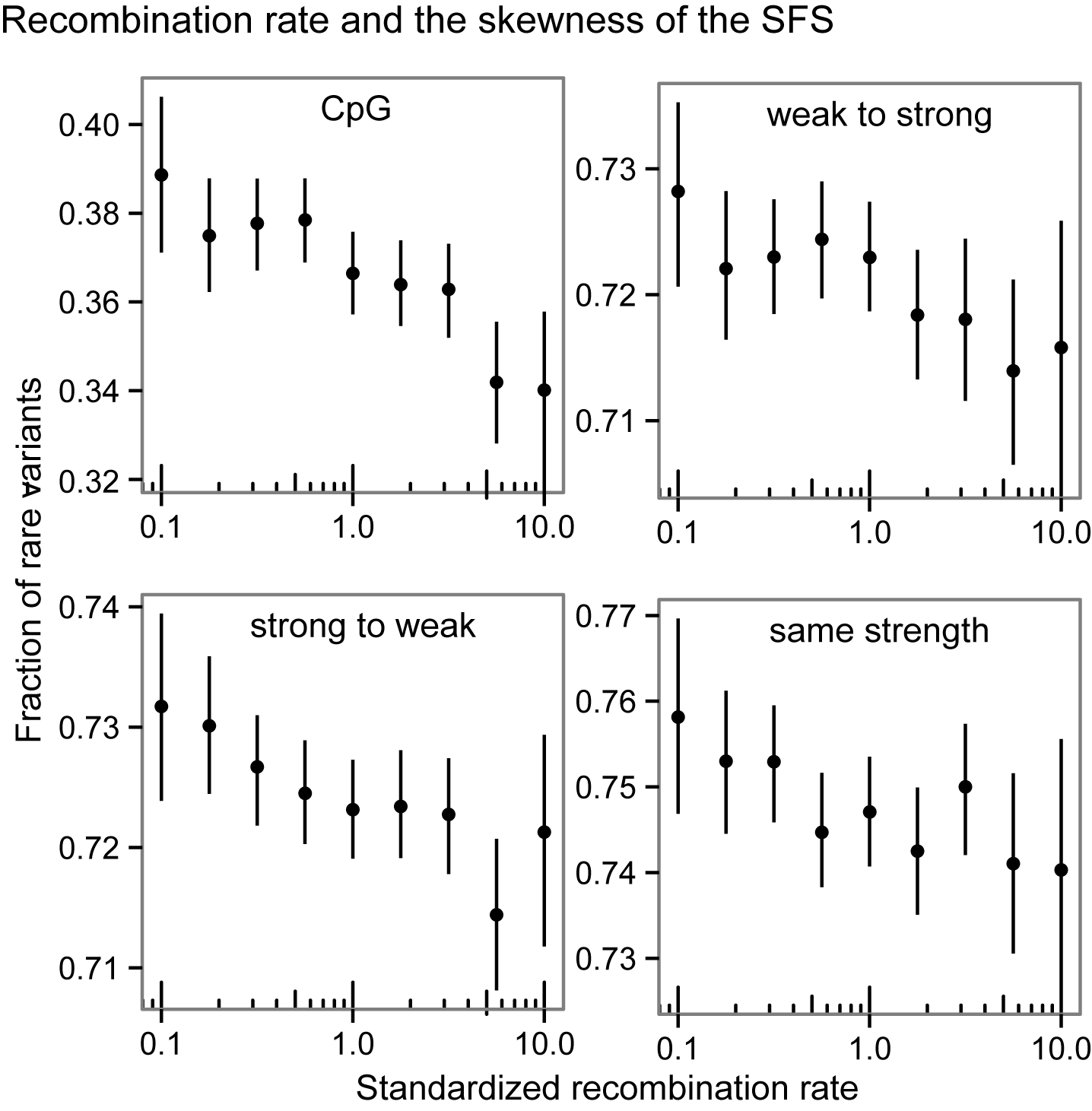

Supplement: S5 Fig — Recombination rate is positively correlated with mutation rate, and is likely driving the negative correlation of recombination rate and the fraction of rare variants. The different panels demonstrate that mutations that are subject to biased gene conversion, as well as those that are not, exhibit a negative correlation between recombination rate and the fraction of rare variants. x-axis values are binned on a logarithmic scale, and are standardized to a genomewide mean. (TIF) [file pgen.1006489.s006.tif]

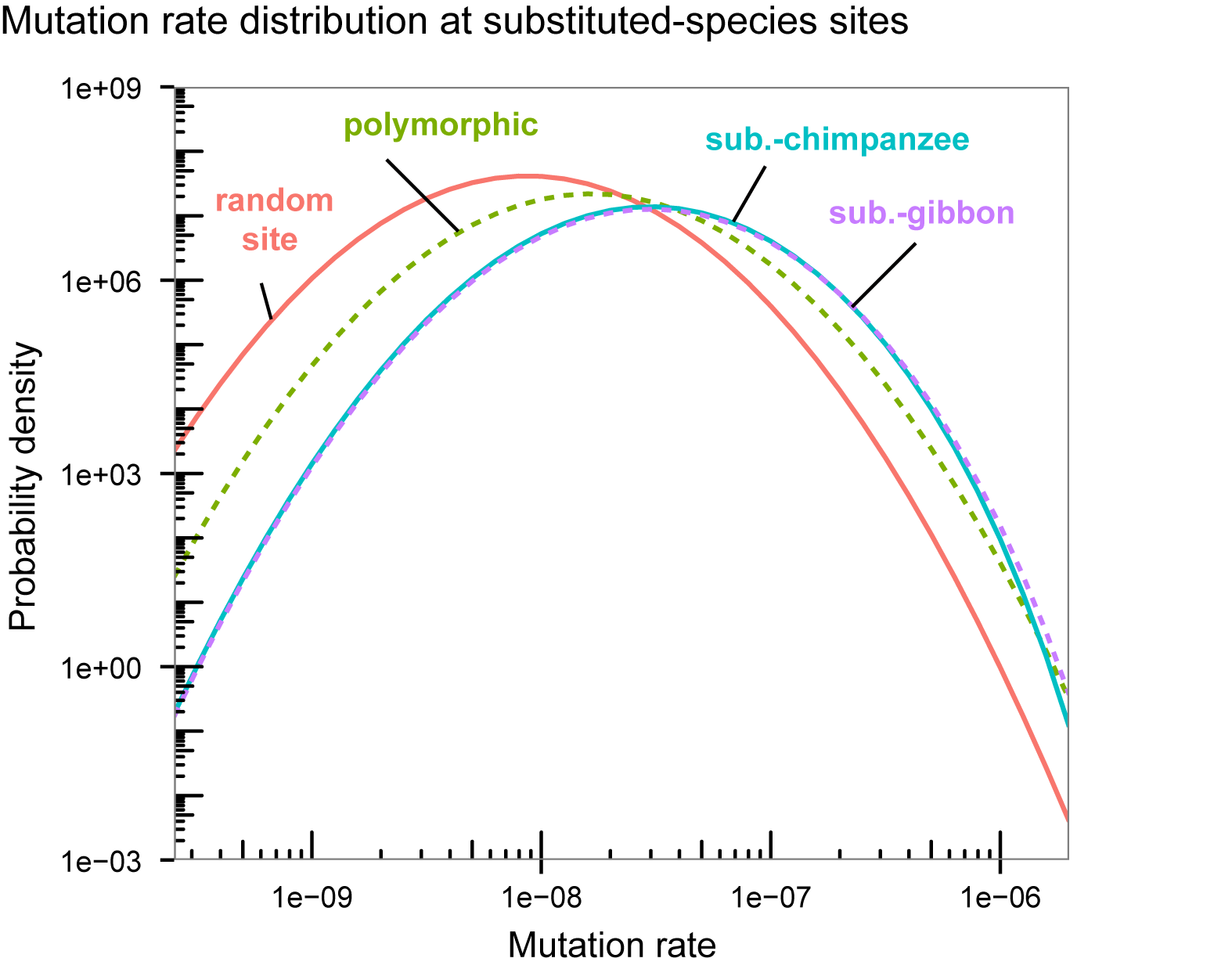

Supplement: S6 Fig — These results were computed using a simple analytic model and a set of realistic parameters. At substituted-species sites, we expect a distribution skewed towards higher mutation rates compared to random sites, or to random polymorphic sites. However, the distribution of mutation rate changes only slightly across substituted species, as exemplified by the purple (substituted-gibbon) and teal (substituted-chimpanzee) lines. (TIF) [file pgen.1006489.s007.tif]

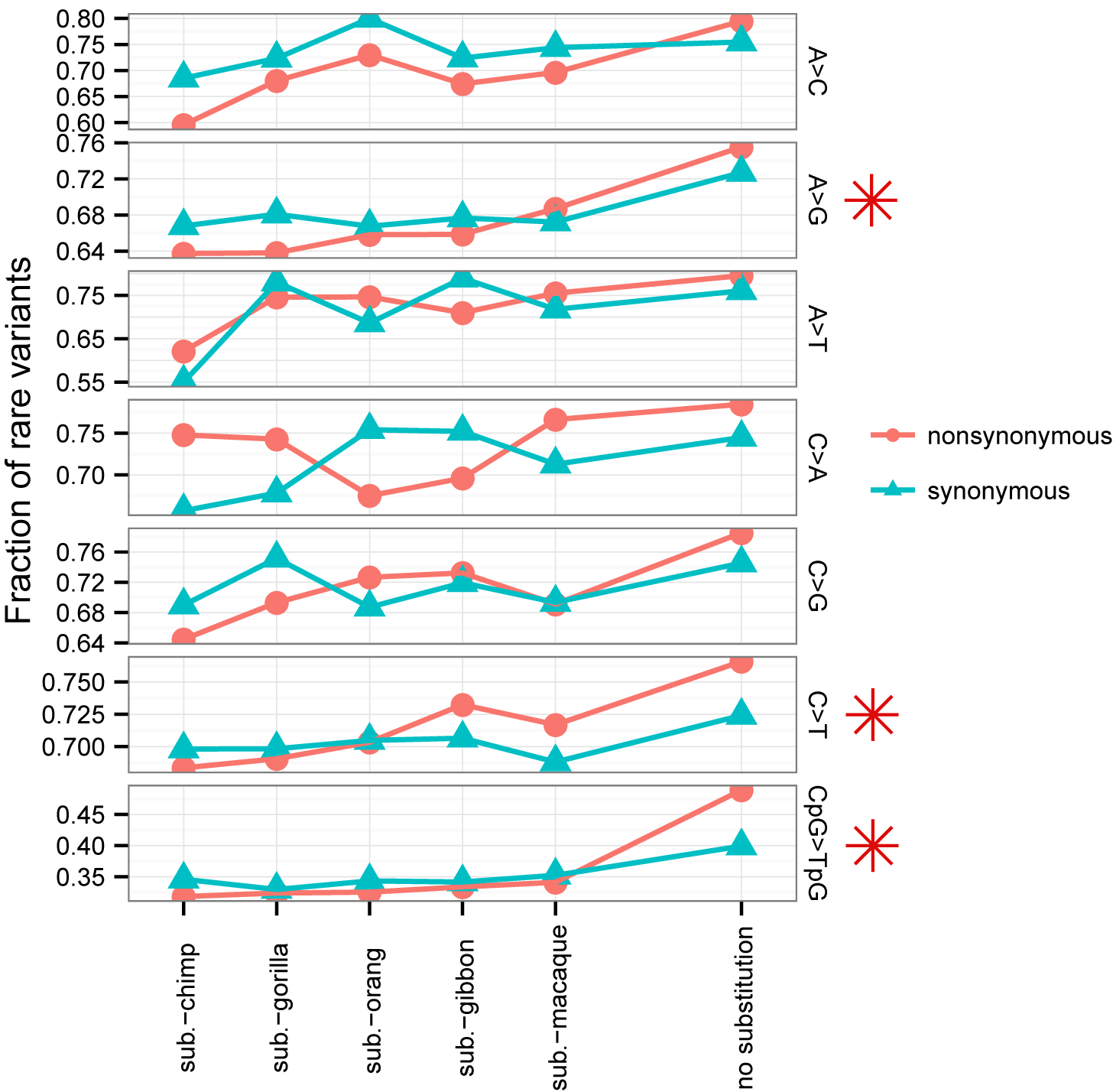

Supplement: S7 Fig — This figure summarizes the same analysis as in the inset of Fig 2C, stratified by non-CpG mononucleotide mutation types and CpG. Red stars denote nonsynonymous trends that exhibited significant Spearman correlation at a significance level of 10%. (TIF) [file pgen.1006489.s008.tif]

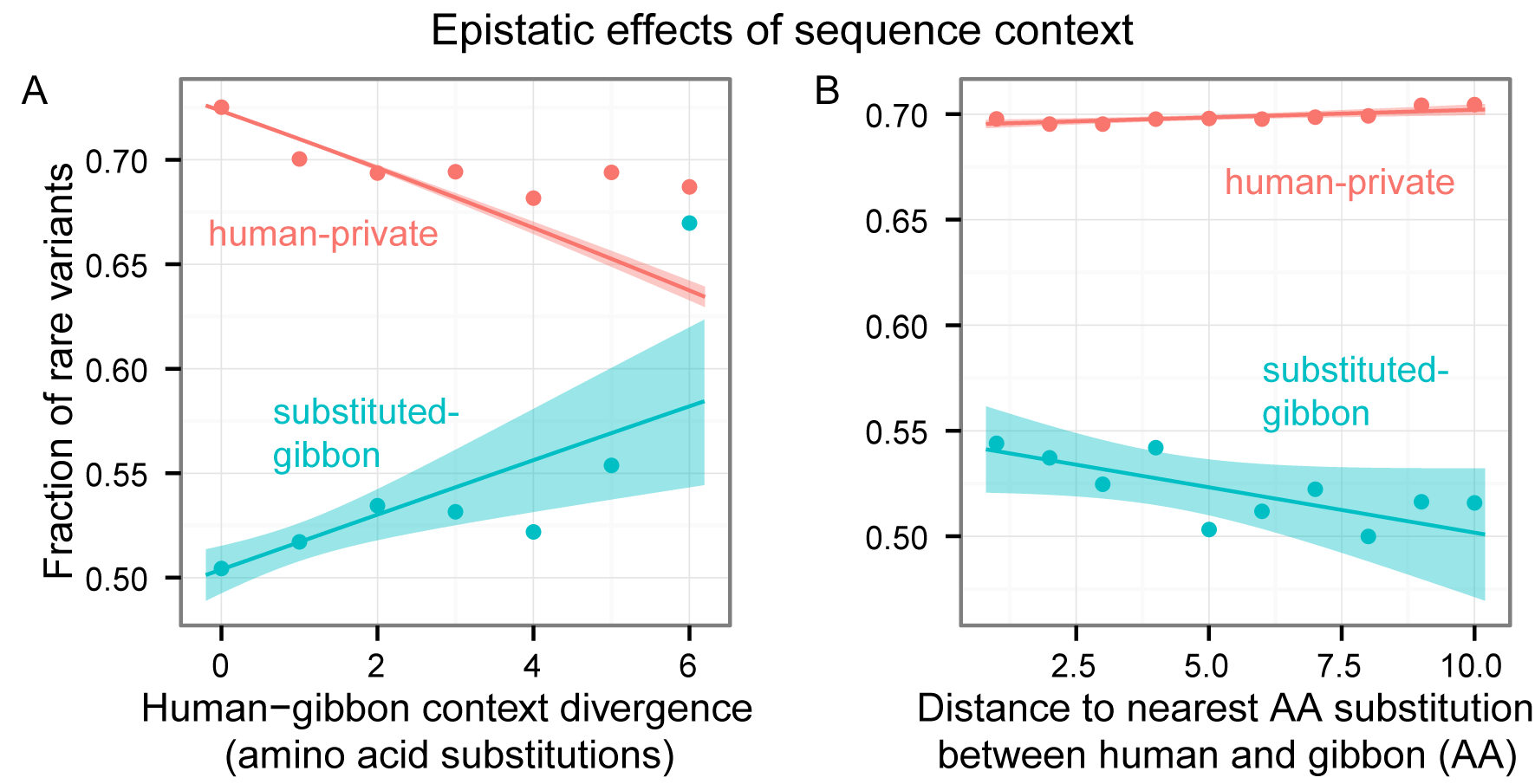

Supplement: S8 Fig — Dots show means. Lines and shaded regions show simple logistic model fits to the data and associated confidence bands. The more diverged the sequence context in the substituted-species is from humans, the higher the fraction of rare variants. The two panels exhibit the same trend with different measures of sequence context divergence in a window of 9 residues upstream and 9 downstream of the SNP. In human-private sites, the trend is reversed: the fraction of rare variants decreased with sequence context divergence from gibbon, consistent with higher regional mutation rates and lower constraint implied by higher sequence context divergence. (TIF) [file pgen.1006489.s009.tif]

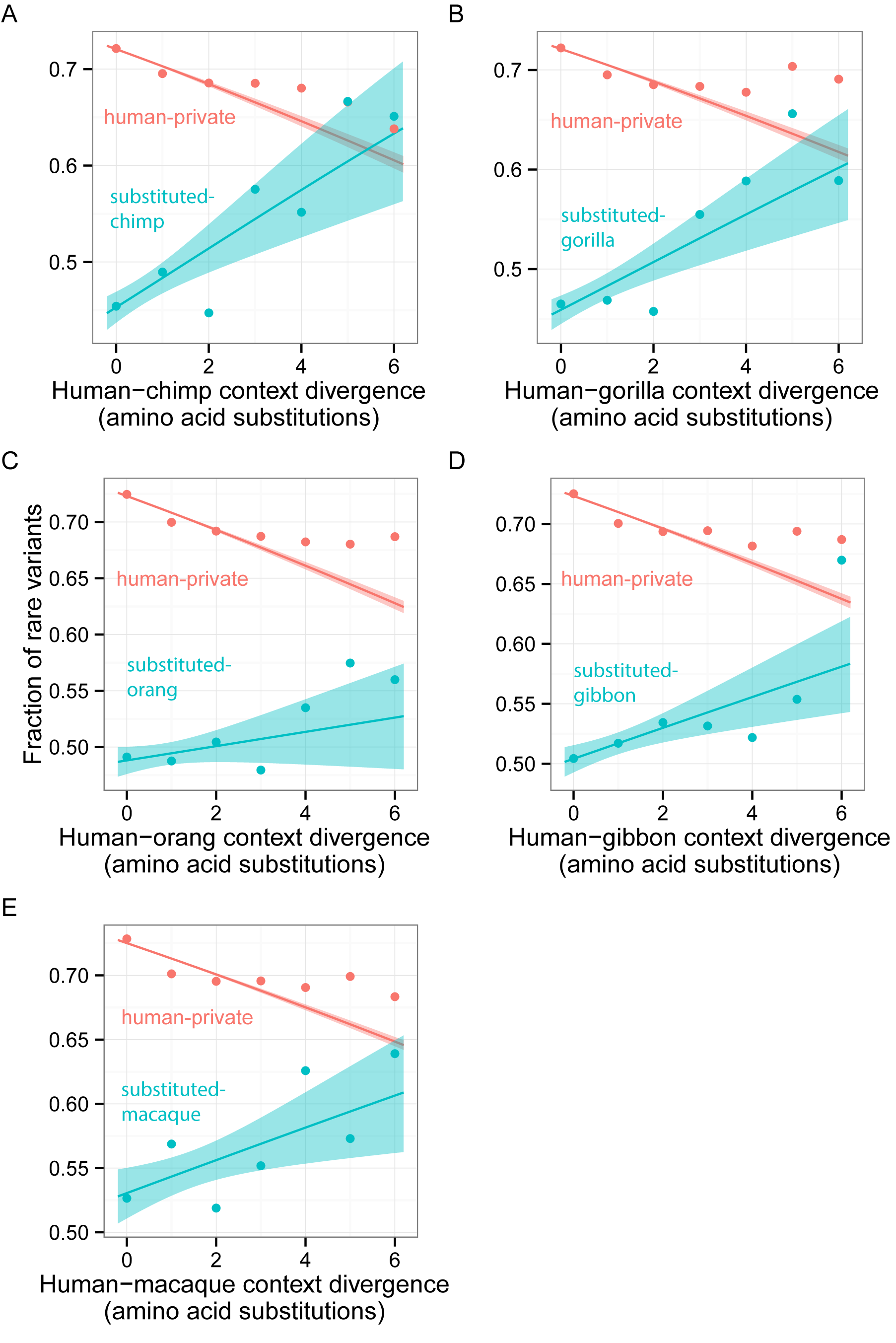

Supplement: S9 Fig — Dots show means. Lines and shaded regions show predictions of a simple logistic model fits to the data and their associated confidence bands. The more diverged the sequence context in the substituted-species is from humans, the higher the fraction of rare variants. Sequence context divergence is calculated as the number of amino acid substitutions in a window of 9 residues upstream and 9 downstream of the SNP. In human-private sites, the trend is reversed: the fraction of rare variants decreases with sequence context divergence from the substituted species, consistent with higher regional mutation rates and lower constraint implied by higher sequence context divergence. (TIF) [file pgen.1006489.s010.tif]

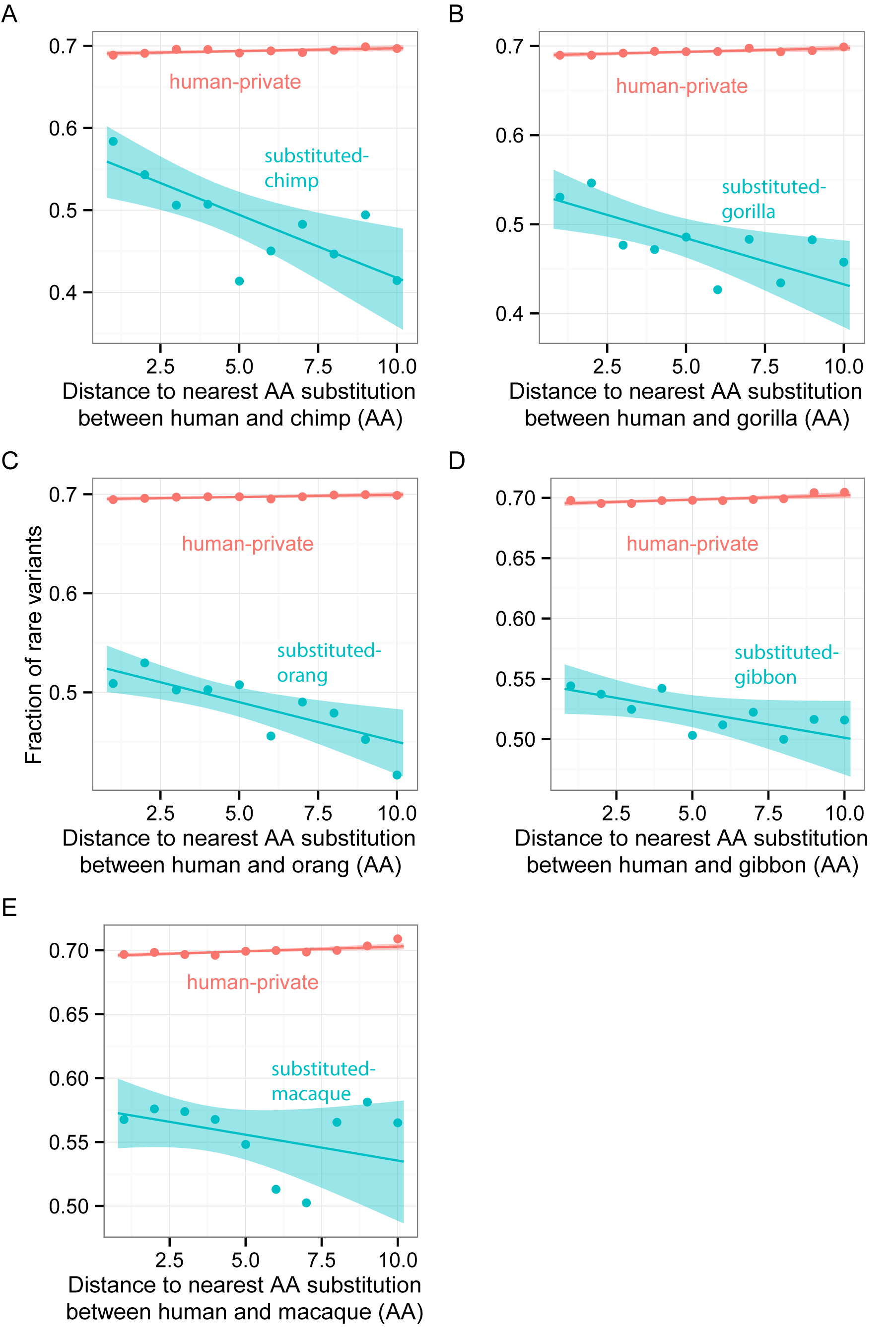

Supplement: S10 Fig — Dots show means. Lines and shaded regions show predictions of a simple logistic model fits to the data and their associated confidence bands. The more diverged the sequence context in the substituted-species is from humans, the higher the fraction of rare variants. Sequence context divergence is calculated as the distance to the nearest amino acid substitutions in a window of 9 residues upstream and 9 downstream of the SNP. (TIF) [file pgen.1006489.s011.tif]

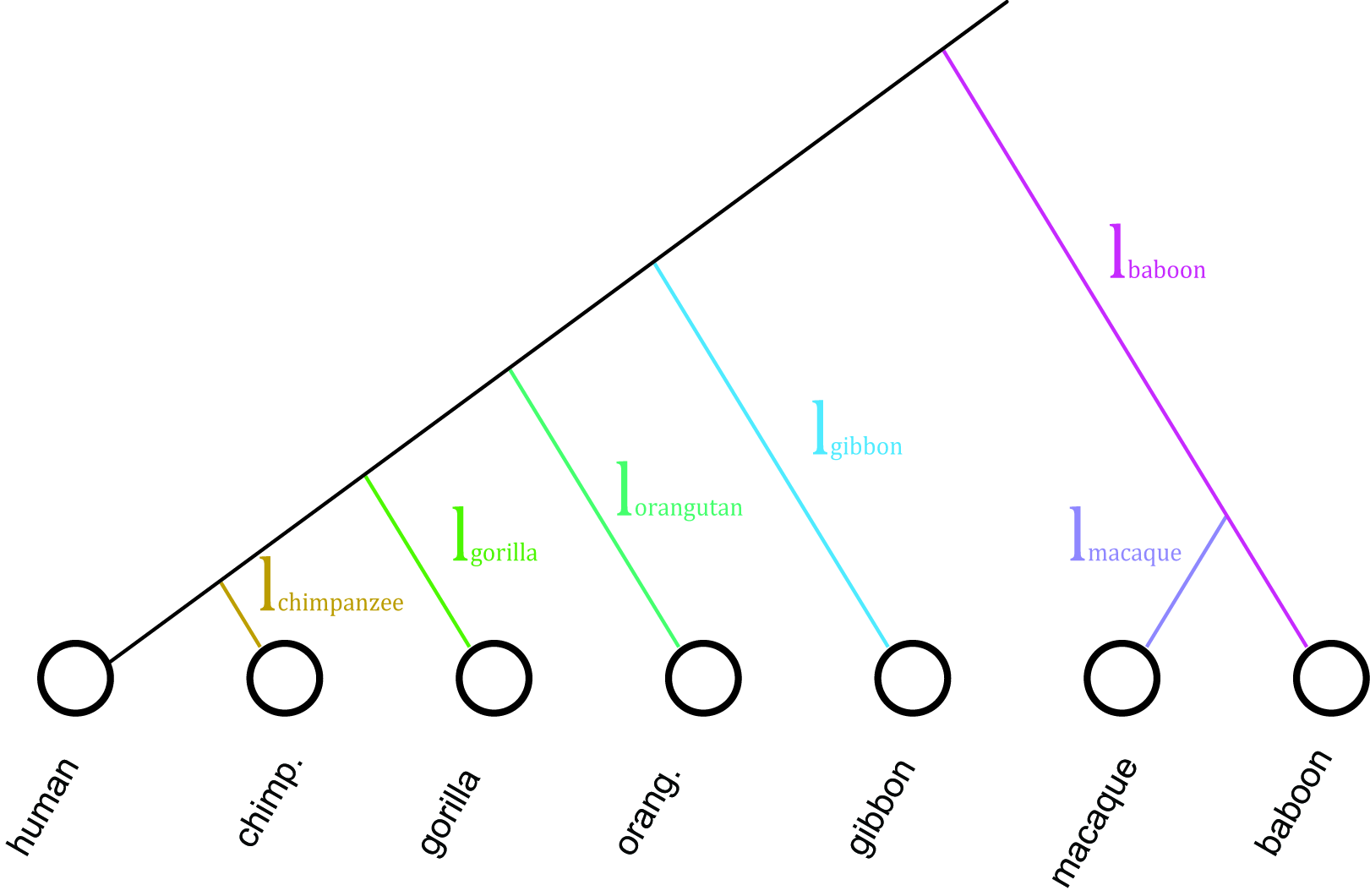

Supplement: S11 Fig — (TIF) [file pgen.1006489.s012.tif]

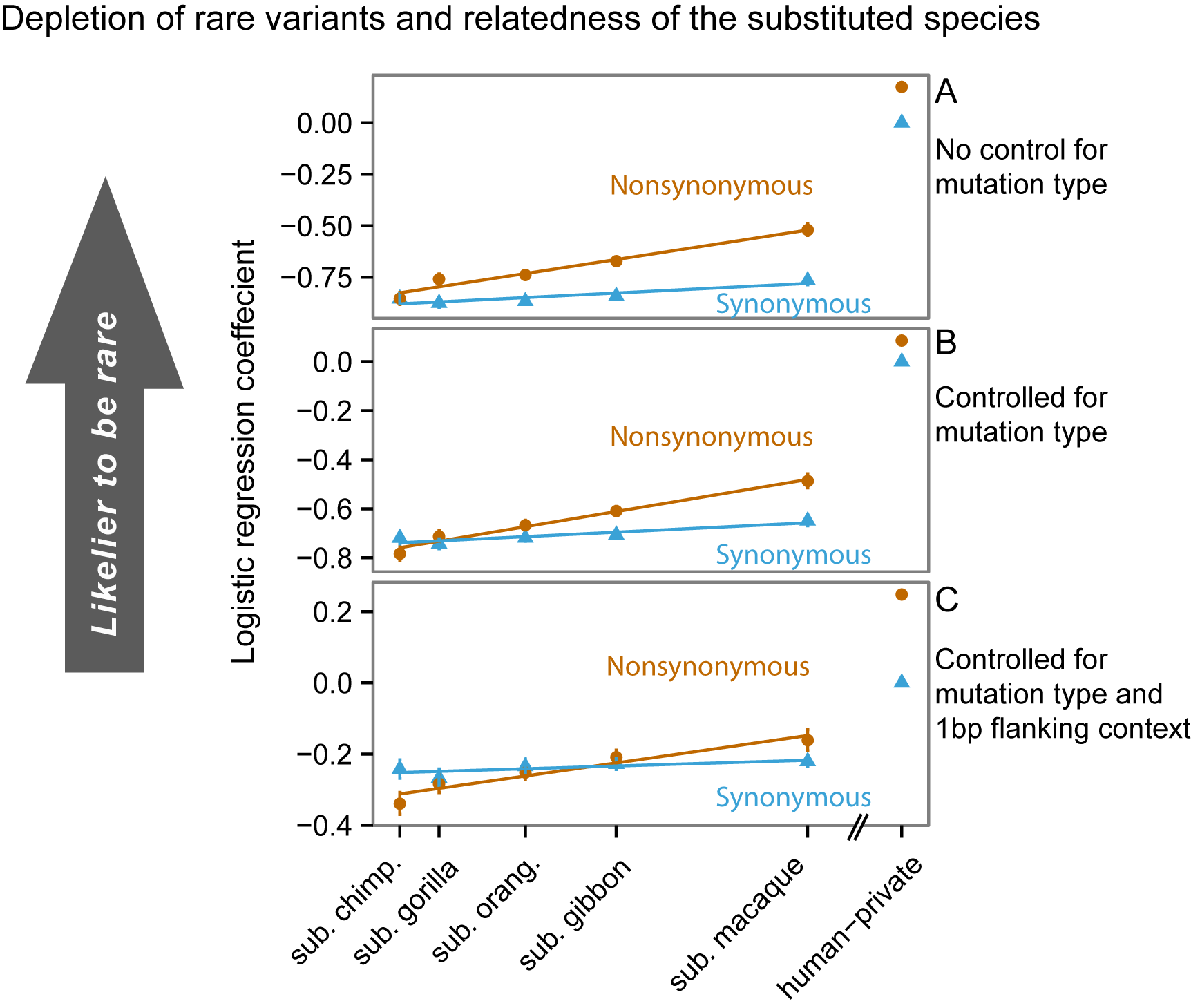

Supplement: S12 Fig — Like Fig 6, this figure shows logistic regression coefficient estimates and their corresponding standard errors. Here, however, CpG transitions were included in the analysis. Substituted-species labels are spaced by their split times from humans. The lines are the least-squares line fitting the coefficients to the split times. (A) Estimates from a simple logistic regression to the substituted species. The trend is partly due to mutational composition differences between substituted-species categories. To test whether the trend is driven solely by mutational rate differences, we estimate coefficients in a model including the variation explained by (B) mononucleotide mutation type, and (C) combinations of focal mononucleotide mutations and upstream and downstream nucleotides. Even after controlling for mutational composition with these models, a significant trend persists for nonsynonymous variants. (TIF) [file pgen.1006489.s013.tif]
